# Universal preimplantation genetic testing for monogenic disease (Karyomapping): diagnosis of >1000 unique disorders with no detected misdiagnoses

**DOI:** 10.1093/humrep/deaf198

**Published:** 2025-10-26

**Authors:** Alessia Schadwell, Olivia Whiting, Leoni Xanthopoulou, Pere Colls, Evangelia Bakosi, N-Neka Goodall, Lia Ribustello, Peter Ellis, Tony Gordon, Darren K Griffin

**Affiliations:** School of Natural Sciences, University of Kent, Canterbury, UK; School of Natural Sciences, University of Kent, Canterbury, UK; CooperSurgical, London, UK; CooperSurgical, Livingston, NJ, USA; CooperSurgical, London, UK; CooperSurgical, Livingston, NJ, USA; CooperSurgical, Livingston, NJ, USA; School of Natural Sciences, University of Kent, Canterbury, UK; CooperSurgical, London, UK; School of Natural Sciences, University of Kent, Canterbury, UK; Preimplantation Genetics Group, EGA Institute for Women’s Health, University College London, London, UK

**Keywords:** karyomapping, PGT-M, universal diagnosis, PGT, IVF, embryo biopsy, SNP chips, haploblocks, misdiagnosis

## Abstract

**STUDY QUESTION:**

Can a universal diagnostic test (Karyomapping) be applied for preimplantation genetic testing for multiple monogenic disorders (PGT-M) and what is the misdiagnosis rate?

**SUMMARY ANSWER:**

Among 9020 cases of PGT-M, >1000 different disorders were diagnosed by Karyomapping; independent validation of >70% of cases did not detect a misdiagnosis.

**WHAT IS KNOWN ALREADY:**

PGT-M, first performed in 1992, has been used for ∼40 000 clinical cases worldwide. A limiting factor in direct testing for disease mutations, however, is the need to design assays specific for each affected allele. Karyomapping, based on haplotype phasing using SNP microarrays, was developed in 2010 as a single, method tracing inheritance of any monogenic disorder. Karyomapping eliminates the impact of allele drop-out and DNA contamination on test accuracy and facilitates a short work-up time as the same assay platform is used for every case.

**STUDY DESIGN, SIZE, DURATION:**

Here, we used Karyomapping on a large PGT-M series from one diagnostic base from January 2014 to December 2021.

**PARTICIPANTS/MATERIALS, SETTING, METHODS:**

The 9020 individual Karyomapping cases were performed in three CooperSurgical genetic testing laboratories, in Livingston NJ, Michigan, or London (UK). All cases involved trophectoderm biopsy with embryo vitrification. DNA from cheek brush samples was obtained from both parents and an affected reference family member where possible. Genomic DNAs and that of whole genome amplified DNA from embryo biopsies were subjected to SNP microarray. Karyomapping was performed according to manufacturer’s instructions by first importing into BlueFuse Multi software. Inheritance was determined as to where at-risk allele(s) were inherited, with 10 supporting 5′ and 3′ Key SNPs in a 2 Mbp flanking window. Wherever possible, direct mutation testing was performed using Sanger sequencing.

**MAIN RESULTS AND THE ROLE OF CHANCE:**

A total of 1017 unique disorders were detected from mutations in 912 genes. Validation of 4120 mutations was possible in 73% of cases by direct sequencing, which confirmed that all diagnoses that could be assayed were accurate.

**LIMITATIONS, REASONS FOR CAUTION:**

Karyomapping can be limited by the availability of a reference, as well as parental genomic DNA, and some loci near the telomere may be more difficult to detect because of the limitations of the SNP array rather than the Karyomapping algorithm. Of the 27% of cases where we could not confirm the findings, we cannot comment on the misdiagnosis rate.

**WIDER IMPLICATIONS OF THE FINDINGS:**

Karyomapping is now the single most used approach for PGT-M. As new approaches increasingly involve DNA sequencing, PGT for all genetic disease becomes possible by encapsulating the principles of Karyomapping and incorporating chromosome copy number analysis.

**TRIAL REGISTRATION NUMBER:**

N/A.

**STUDY FUNDING/COMPETING INTEREST(S):**

This research was funded by CooperSurgical. The PhD programs of A.S. and O.W. were supported by CooperSurgical (paid to institution). A.S. has received travel support and IT equipment from CooperSurgical. O.W. has received travel support and provision of a company laptop from CooperSurgical. L.X., P.C., E.B., and T.G. are employees of and hold stock/share ownership in CooperSurgical. N.-N.G. is an employee of, has received meeting registration fees from, and holds stock/share ownership in CooperSurgical. L.R. is an employee of CooperSurgical. D.K.G. has received consulting fees and travel support from CooperSurgical. P.E. has nothing to declare.

## Introduction

Preimplantation genetic testing for monogenic disorders (PGT-M) was first reported by Handyside *et al.* (1992). Contemporary figures on the total number of worldwide cases to date present a challenging calculation, since consortium data collection ended around 2018, with 1388 PGT-M cases being reported in that year (Spinella *et al.*, 2023). The first PGT consortium (ESHRE PGD Consortium Steering Committee, 1999) collated all reported cases to 1998 (200 at the time), with figures of generally between 600 and 700 per year thereafter up until 2006. The year 2007 saw a rapid rise, when case numbers per year of ∼1300–1800 were reported up until 2013–2014 (Girardet *et al.*, 2018), then peaked at around 2750 per year in 2015 (for full list of ESHRE PGT consortia reports, see https://www.eshre.eu/Data-collection-and-research/Consortia/PGD-Consortium/PGD-Consortium-Publications). Rough calculations of worldwide cases up to and including 2018 are thus around 25 000 in total reported, more likely nearer 30 000 when we consider that not all practicing clinics reported to the consortium. If we then extrapolate a total of 1300–2500 cases per year in the years 2019–2024, eliminating 1 year (half of 2020 and half of 2021) because of the Covid pandemic and lockdown, then a reasonable estimate is that the cumulative number of worldwide PGT-M cases will approach 40 000 in 2025.

The technology used for diagnosis has changed, variously involving nested PCR, fluorescent PCR, whole genome amplification (WGA), direct mutation analysis, haplotype mapping, next generation sequencing (NGS), and variants of similar technologies (De Rycke and Verdyck, 2020; Hardy, 2020). The overall PGT-M approach has, however, perpetually been challenged by the issues of allele dropout (i.e. only one allele amplifying from a heterozygous embryo) and DNA contamination (e.g. of maternal or operator origin), both of which can lead to misdiagnosis (false positives and false negatives) (De Rycke and Verdyck, 2020). A widespread switch in clinical practice from cleavage stage and/or polar body biopsy to trophectoderm biopsy (of Day 5–6 blastocyst embryos) minimized allele dropout, given that 5–10 cells became available for analysis rather than one (De Rycke and Verdyck, 2020). Moreover, when direct mutation analysis was combined with haplotype analysis, both the disease allele itself was identified, as were markers either side of the locus of interest. This established whether the haploblock containing the disease allele had been inherited, thereby generating confirmatory approaches for establishing disorder status. By establishing the meiotic phase, determined through comparison with a family member of known genotype (often an older, affected child), molecular markers (typically hypervariable short tandem repeats (STRs)) were used to reduce the chances of misdiagnosis through allele dropout or DNA contamination significantly (De Rycke and Verdyck, 2020).

Karyomapping, based on haploblock analysis of genome-wide markers, was first developed in 2010 as a comprehensive means of PGT, demonstrating efficacy in detecting most monogenic disorders as well as meiotic trisomy, uniparental disomy (UPD), whole and partial chromosome loss and human leukocyte antigen (HLA) matching (Handyside *et al.*, 2010; Griffin and Gould, 2017). Although originally developed as a platform-independent algorithm that could use either single-nucleotide polymorphism array (SNP chip) data or raw sequence data, since its adoption, Karyomapping has exclusively used an Illumina SNP chip of increasing complexity. In addition to the above-mentioned advantage of haploblock analysis over direct mutation testing, the key advantage of Karyomapping over earlier methodologies is that individual locus-specific ‘work-up’ involving the design and validation of multiple STRs need not be performed. Therefore, the speed to first diagnosis has the potential to be improved relative to STR-based haplotyping methods.

Karyomapping’s first proof of principle clinically for Smith–Lemli–Opitz and Marfan Syndromes (Natesan *et al.*, 2014a; Thornhill *et al.*, 2015) led to the reporting of the first set of cases (Natesan *et al.*, 2014b) in which the accuracy of targeted haplotyping combined with direct mutation detection was compared with that of Karyomapping. In that study, 213/218 (97.7%) samples from 44 PGT cases comprising 25 single-gene defects (several modes of inheritance) proved concordant in 208 (97.7%) samples. The remaining five non-concordant cases were due to consanguineous regions leading to limited or inconsistent haplotyping outputs. At the time, it was suggested that Karyomapping could facilitate the detection of almost any monogenic disorder, or combination of loci, in single cells. This thereby expanded the conditions for which PGT-M could be offered to patients without the need for customized test development (Natesan *et al.*, 2014a,b; Thornhill *et al.*, 2015).

Despite the fact that Karyomapping can be performed for PGT-M without further testing, it may nonetheless be combined with direct mutation detection to give additional confirmatory diagnosis (see also the Discussion section), particularly if the laboratory can ensure no delay in workup time (typically because the mutations have been detected previously and thus an allele-specific assay is already designed). Relatively recent publications reported a small case series run by Karyomapping with added direct mutation analysis to improve test accuracy (Ben-Nagi *et al.*, 2017).

While there are other linkage-based approaches, including haplarithmisis (Zamani Esteki *et al.*, 2015, marketed as ‘PGT-One’ by Agilent) and others (Chen *et al.*, 2021; Xie *et al.*, 2022; Hornak *et al.*, 2024), Karyomapping is, to the best of our knowledge, the most widely practised global haplotyping technology used for PGT-M. Most cases have been performed by CooperGenomics, with 200 IVF clinics being serviced by two diagnostic laboratories. Newer sequence-based methods (Janssen *et al.*, 2024) also now use the principles of Karyomapping for haploblock detection. In brief, the Karyomapping (and haplarithmisis to some degree) initially restricts analysis to informative SNPs, i.e. those where one parent is heterozygous and another homozygous, and then determines phasing in each embryo using so-called ‘key SNPs’. Key SNPs are those that are detected as heterozygous in the embryo, thus ensuring the results are not confounded by allele dropout in the embryo (Handyside *et al.*, 2010; Handyside, 2015). Haplotyping by STR markers, Karyomapping, haplarithmisis, and haplotyping by sequencing all take advantage of whole genome amplification technologies, through which multiple loci can be assessed from only a 5–10 cell template. Most recently, a new form of WGA, termed primary template directed amplification (PTA) (Weier *et al.*, 2024), demonstrated improved genomic coverage and SNP allelic balance than multiple displacement amplification (MDA), which is traditionally used for Karyomapping, and may be able to improve haplotyping accuracy.

The purpose of the current study is to establish whether the potential to use Karyomapping as a universal means of PGT-M has now been realized. There has not yet, however, been a comprehensive overview of the capabilities of this technology in terms of its accuracy and range of disorders it can diagnose. With the above in mind, we now report the largest series of Karyomapping cases to date, with concurrent direct mutation analysis for most treatment cycles where possible. We demonstrate the comprehensive nature of genome-wide linkage analysis for PGT-M, reporting, for the first time, over 1000 different disorders detected using a single test. The mutations for each disease, the inheritance type for each case, the percentage of successful analysis, and the overall misdiagnosis rate are all reported, along with follow-up statistics such as call rate and allele drop-out rate.

## Materials and methods

### Participant identification and data collection

A total of 9020 individual Karyomapping cases were performed over the time period of January 2014 to December 2021. The cases in this series were performed in three CooperSurgical genetic testing laboratories, in Livingston NJ, Michigan, or London (UK). Data were collected as part of normal laboratory service and not part of a clinical study. CooperSurgical data governance procedures were reviewed by the Central Research Ethics Advisory Group at the University of Kent, and the project was approved as a retrospective analysis of anonymized consented clinical data (CREAG057-04-024). For all cases, patient and family history was taken by a laboratory genetic counsellor and mutation reports were collected from accredited external labs. Cases in the UK were accepted when the disorder was found to be on the HFEA list of approved conditions. Outside the UK, an internal CooperSurgical review board accepted cases in line with the ASRM guidelines for ‘Indications and management of preimplantation genetic testing for monogenic conditions: a committee opinion’ (Practice Committee and Genetic Counseling Professional Group of the American Society for Reproductive Medicine, 2023).

### Laboratory protocols

All cases in this series involved trophectoderm biopsy, with embryo vitrification, not requiring fresh embryo transfer. Where possible, cheek brush samples were obtained from an affected reference family member and DNA was extracted. DNA samples of family member were subjected to SNP microarray (Illumina, 2018) and a ‘case preparation’ was performed to ensure that there would be sufficient 5′, intragenic, 3′ informative SNPs to allow PGT-M to proceed. Once the family history, mutation report, and cheek brush swabs were obtained, case reports were prepared generally in less than 8 weeks.

Embryo biopsies were collected in a CooperSurgical (Trumbull, CT) biopsy kit and shipped to either Livingston (NJ) or London (UK). Three amplification strategies were used, either MDA, Repli-g Single Cell kit (Qiagen, Venlo, USA), or PicoPlex (Takara Bio, USA) according to the manufacturer’s instructions. Karyomapping (VitroLife, Sweden) was performed according to the manufacturer’s instructions. Briefly, amplified DNA was applied to Illumina CytoSNP12 Karyomapping arrays using the Infinium assay protocol (Illumina, San Diego, USA). Results from the parents and affected reference such as grandparent or affected child (where available) were imported in BlueFuse Multi. Inheritance was determined as to where at-risk allele(s) were inherited, with 10 supporting 5′ and 3′ Key SNPs in 2 Mbp flanking window (Natesan *et al.*, 2014b). If insufficient 5′ and 3′ Key SNPs were found within a 2 Mbp flanking window, usually for sub-telomeric disorders, the flanking window was increased to 10 Mbp, or flanking STR were used to supplement phasing. In these analyses, MDA was only used for triplet repeat expansion disorders (e.g. fragile X). Internal Cooper data suggest that SNP call rates, allele dropout (ADO) rates, and the average number of key SNPs within the 2 Mb window are marginally higher using MDA, and the statistics for MDA are slightly better than PicoPlex, however we routinely used PicoPlex for all but the triplet repeat expansion cases as it allows us to perform PGT-A simultaneously (which MDA does not). Similarly, PicoPlex is not compatible with detection of triplet repeat disorders.

### Direct mutation analysis

Subsequently, wherever possible, direct mutation assays were also prepared for mutations that were amenable to direct mutation testing. Briefly, the protocol involved designing primers to target and amplify the region of interest for each mutation tested by PCR for downstream testing via mini-sequencing, Sanger sequencing, or Fragment Analysis on an ABI 3130, 3730, 3500, or SeqStudio 24 Flex Genetic Analyser. Data interpretation was performed manually by lab scientists in conjunction with the SNP linkage analysis interpretation. SNP linkage analysis is the primary result used for phasing, and direct mutation testing results are utilized as confirmatory testing. Root cause protocols involved the re-running of all references, plus re-analysis of the Sanger direct mutations, and full re-analysis. If the root cause was not established, then further reference samples were obtained and tested and STRs were used to supplement the Karyomapping results. The main cause of non-conformity was either sample swap (correct parents, incorrect embryo transferred) or natural conception, although both were extremely rare <10 cases. Maternal cell contamination, the most common (<0.03% of cases CooperSurgical internal data) form of PGT-A misdiagnosis, was identified by Karyomapping.

### Analysis of non-conformities

If a non-conformity was reported in PGT-M results, follow-up root cause investigation was performed. DNA from foetal/neonatal/children was collected and applied to the Karyomapping SNP array process to determine the root cause, looking for matching/non-matching of haploblocks to embryos of the PGT-M cycle as follows. In the event that the laboratory received a discrepancy report from a clinic after the transfer of an embryo based on the PGT results reported, this was logged by the laboratory as a non-conformity, and the genetic report confirming the discrepancy was requested. The archived data of the original PGT-M results were reviewed to confirm that they were consistent with the original results reported and that the quality control data of the original analysis were within acceptable limits. For the embryo that was transferred, the stored whole genome amplified product, contained in the original tube provided by the referring centre, was then reanalyzed to confirm that the rerun result was concordant with the original result reported, thereby ruling out an error at the PGT laboratory. Additional tests to identify the source of the discrepancy were then proposed, requiring a DNA sample from the pregnancy that originated from the embryo transfer. The DNA from patient, partner, pregnancy DNA, and original embryo biopsy were run on the PGT-M SNP linkage analysis platform to confirm that the pregnancy originated from the transferred embryo, to rule out potential DNA contamination, natural conception, or the potential transfer of a different embryo or an embryo not belonging to the couple.

## Results

### Overall numbers of disorders, genes, and mutations tested for PGT-M

**Figure 1.**
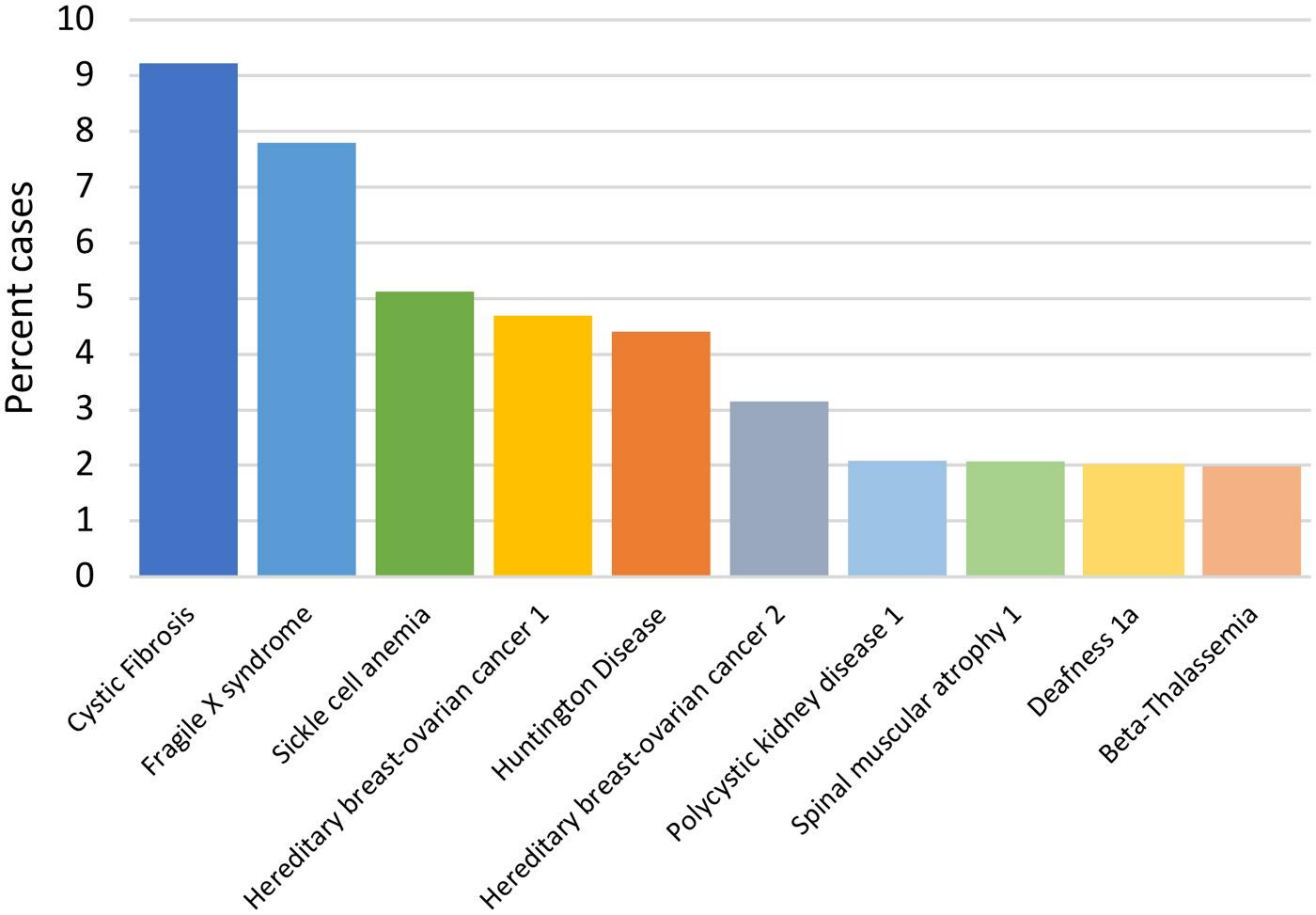
**The 10 most common disorders approved for Karyomapping between 2014 and 2021.** Disorders are represented as a percentage of the total number of cases (n = 9020).

We present here the results of ∼68 000 embryo biopsies from 14 606 IVF cycles comprising 9020 individual cases. These collectively account for 9324 indications, since some cases had multiple indications for PGT. Of these, a total of 234 indications (2.5%) were excluded from further analysis due to non-specificity of the disorder and/or mutation, UPD or HLA testing, or due to a lack of an external mutation report. Parental UPD was found in two cases, which prevented BlueFuse Multi from reporting haploblock findings; in these two cases, this UPD was found in the parental references. When this occurred, then BlueFuse reported an error and no further processing could be completed. The UPD in the reference sample did not have to be in the chromosome carrying the gene of interest since the BlueFuse version at the time of this study, was halted if any UPD in any chromosome in a parental sample was found. After exclusions, this study encompassed 9020 cases with 9090 indications tested for between 2014 and 2021. These in turn comprised 1017 unique disorders (Supplementary Table S1) involving 912 unique genes in this cohort. Of the 1017 individual disorders subject to PGT-M, Cystic Fibrosis was the most commonly tested (832 cases), followed by Fragile X Syndrome (703 cases) (Fig. 1). Other disorders were rarer, with 465 being tested only once.

### Karyomapping expands the spectrum of testable mutations for PGT-M

Across the 9020 cases, a total of 912 genes were tested, with the associated mutations ranging from single-base pair changes to large deletions covering multiple exons. All smaller mutations involving <300 base pairs were eligible for both linkage analysis and subsequent downstream direct mutation testing. Larger mutations were not eligible for direct testing and so were assessed only via linkage analysis. Where multiple mutations were present at the same locus, with at least one eligible for direct mutation testing and at least one eligible for linkage analysis only, a combined approach was taken. In total, 4930 cases (54.7%) presented with small mutations only, 1611 cases (17.9%) used the combined approach, and 2479 cases (27.5%) presented with longer mutations that were not amenable to direct screening. Most commonly, the latter applied to the testing of expansion disorders. Thus, the use of Karyomapping enabled testing of a significant proportion of cases that could not be tested for by direct mutation analysis, demonstrating the power of this technology to provide robust results even in challenging genomic contexts.

The diagnoses in 6541 cases (72.5%) were verified both by linkage and by direct screening. These cases involved 4120 mutations including single-nucleotide changes (69.4%), insertions (2.5%), deletions (20.6%), indels (0.9%), and duplications (6.2%) located across 863 genes. The most common of these mutations are listed in Table 1. The PKD1 gene had the highest number of distinct mutations eligible for direct testing (171 different mutations), followed by the CFTR gene (145 different mutations). The most common individual mutation tested was c.1521_1523delCTT in the CFTR gene, with 620 patients and partners requiring testing for this gene. We are unable to include direct mutation lists in the supplementary information since combined mutation status and disorder identification could potentially deanonymize patients for the extremely rare disorders.

**Table 1.** The 10 most common mutations eligible for direct mutation analysis.

Gene	Mutation	Disease	Number of cases
**CFTR**	c.1521_1523delCTT	Cystic Fibrosis	620
**HBB**	c.20A>T	Sickle Cell Disease	453
**BRCA1**	c.68_69delAG	Breast Cancer	120
**BRCA2**	c.5946delT	Breast Cancer	86
**GJB2**	c.109G>A	Palmoplantar Keratoderma with Deafness	75
**CFTR**	c.350G>A	Cystic Fibrosis	71
**GJB2**	c.35delG	Palmoplantar Keratoderma with Deafness	66
**HBB**	c.19G>A	Sickle Cell Disease	43
**CFTR**	c.3846G>A	Cystic Fibrosis	43
**CYP21A2**	c.844G>T	Congenital Adrenal Hyperplasia	41

### Data analysis statistics

We have recently published data on our Karyomapping cases (Martel *et al.*, 2024) which describes embryo analysis per inheritance pattern in our own (CooperSurgical Karyomapping and NGS-based PGT-M) dataset. Breaking down these patterns is complex since that also includes PGT-A and hence maternal age. Our SNP call rates for PicoPlex SNP calls were approximately 0.70 (0.60 pass rate); for MDA, the rates were higher, i.e. in the 0.70–0.95 range (Natesan *et al.*, 2014a,b). Our test aim was always to have ≥10 informative Key SNPs per biopsy across the 2 Mbp surrounding windows across the gene of interest. Where possible direct Sanger mutation sequencing for the case mutation was performed. A minimum of seven informative Key SNPs after PicoPlex WGA or six informative Key SNPs after MDA WGA across the windows and gene of interest was our standard protocol, if direct mutation sequencing and/or intragenic SNPs were reported in a biopsy test. Windows surrounding the gene of interest could be expanded from 2 Mb per side to up to 5 Mbp in cases of reduced informativity (typically consanguineous cases or in the case of genes with poor SNP coverage) and when a reduced accuracy (due to the higher risk of recombinations) consent form was signed by patients after appropriate genetic counseling. Recombination rates that led to inconclusive results were <1% whether picoplex or MDA was used for WGA. In terms of ADO rates, these have been validated on previous datasets and were <0.5 on average. The no-result rate, due to no amplification or SNP results of insufficient quality, was 2.35%.

### Trends in PGT-M testing for disorders, genes, and mutations

**Figure 2.**
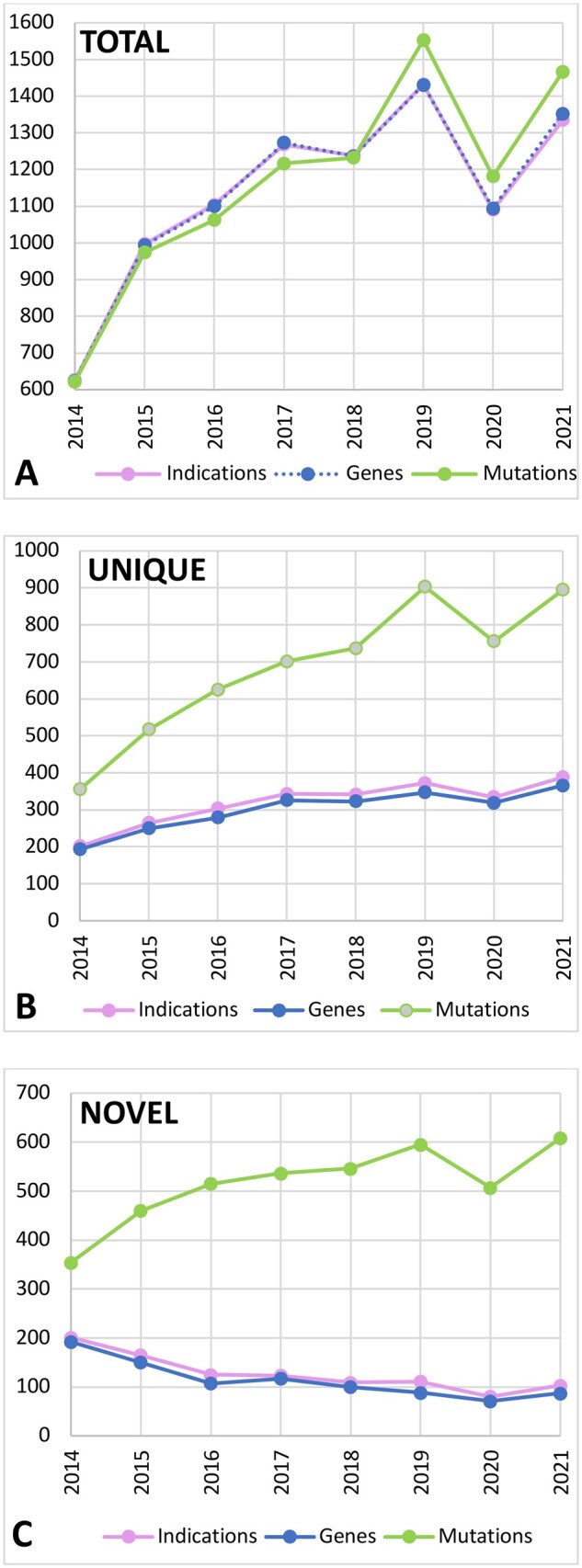
**Annual trends in indications, genes, and mutations between 2014 and 2021.** (**A**) Total number of indications, genes, and mutations observed in each year; (**B**) Number of unique indications, genes, and mutations observed in each year; (**C**) New unique indications, genes, and mutations observed in each year.

Trends were established for the uptake of PGT-M and for disorders tested for (Fig. 2). The biggest category of disorders was those that occurred on a single-case basis, i.e. unique disorders (45.7%), followed by disorders occurring in two to nine cases (41.9%), with only 12.4% of all disorders accounting for 10 or more cases. There were 297 cases that tested for two or more indications.

The number of indications tested for increased on average by 8.2% per year with unique genes concomitantly rising by 8.0% and mutations by 6.2%. Despite a downward trend occurring (−6.9%), an average of 127 disorders novel to PGT-M were added every year (Fig. 2C).

### Trends in PGT-M testing by class of disorder

**Figure 3.**
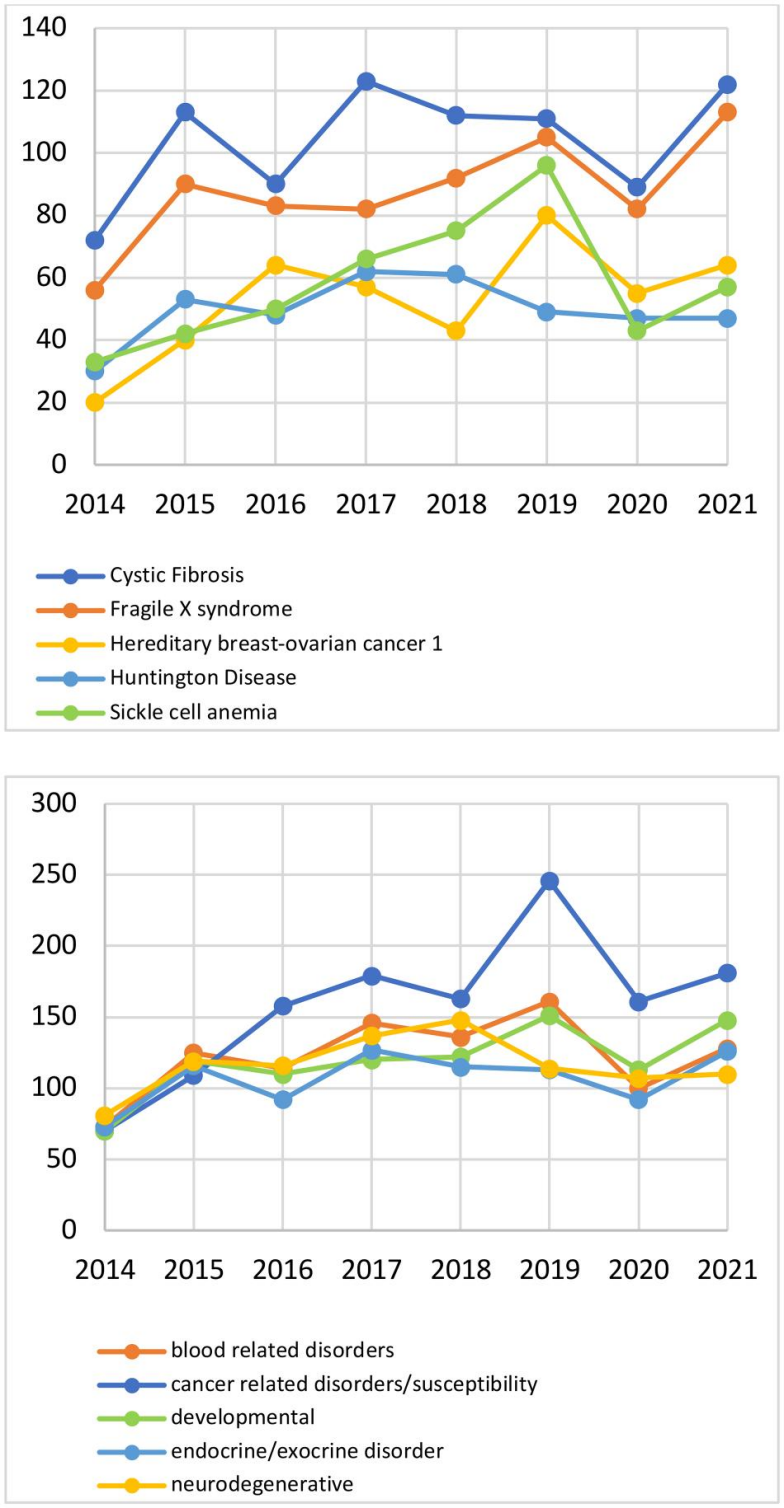
**Annual trends in top five disorders and groups of disorders.** Cases are presented on an annual basis. Overall, the most frequently occurring group was cancer-related disorders (1267 cases; annual growth (AG): 15.3%), followed by blood-related diseases (983 cases; AG: 4.7%), then developmental disorders (953 cases, AG: 7.6%), neurodegenerative diseases, e.g. Charcot-Marie Tooth disease (932 disorders, AG: 1.8%), and finally endocrine/exocrine diseases (854 cases, AG: 3.6%).

The most frequently tested for disorder within the dataset was Cystic Fibrosis (832 cases; annual growth (AG): 3.4%), followed by Fragile X Syndrome (703 cases; AG: 5.2%), then Sickle Cell Anaemia (462 cases; AG: 3.8%), Hereditary Breast and Ovarian Cancer 1 (423 cases; AG: 5.0%), and finally Huntington Disease (397 cases, AG: 1.1%) (Fig. 1). Disorders were grouped into functional groups and trends were established (Fig. 3). Overall, the most frequently occurring group was cancer-related disorders (1267 cases; AG: 15.3%), followed by blood-related diseases (983 cases; AG: 4.7%), then developmental disorders (953 cases, AG: 7.6%), neurodegenerative diseases, e.g. Charcot–Marie Tooth disease (932 disorders, AG: 1.8%), and finally endocrine/exocrine diseases (854 cases, AG: 3.6%). Regarding the unique disorders, most were allocated to the metabolic conditions group, which included 134 unique diseases. This was followed by conditions of the musculo-skeletal system (n = 114), then multisystem disorders (n = 87), those of neurological nature (n = 78), and finally neurodegenerative diseases (n = 72).

### Follow-up: non-conformities reported by clinics

In all analyses of non-conformity by clinics, the root cause analysis indicated no apparent misdiagnoses due to Karyomapping. The main cause of non-conformity was either sample swap (correct parents, incorrect embryo transferred) or natural conception, although both were extremely rare with <10 cases. Maternal cell contamination, the most common (<0.03% of cases CooperSurgical internal data) form of PGT-A misdiagnosis, was identified by Karyomapping.

## Discussion

We provide an evaluation of the scope and range of Karyomapping, demonstrating over 1000 unique conditions diagnosed, representing, to the best of our knowledge, the largest example of a single test being used for a range of disorders for PGT-M. In addition, through contacts in the IVF and genetics field, we estimate that over 1000 IVF clinics worldwide offer Karyomapping, with detection largely serviced by ∼20 diagnostic laboratories. A conservative estimate therefore is that, at the time of writing, ∼30 000 couples or individual patients have benefitted from Karyomapping treatment, with around ∼180 000 embryos biopsied. As such, Karyomapping is currently likely to be the most used diagnostic tool in PGT-M at present, largely because of its applicability to a range of disorders, ease of use, and rapid workup time. The reliability and success of PGT-M in general, and Karyomapping in particular, are very dependent on high-quality data and analysis statistics. The approaches herein described provide that data.

The most recent ESHRE PGT consortium data from 2018 suggested that the most common biopsy stage is still on Day 3 (65% of cases) (Spinella *et al.*, 2023), with trophectoderm biopsies only performed in 33% of cases. We believe this to be out of date, however, and understand that trophectoderm biopsy has largely superseded all other forms of sampling worldwide, with embryological improvements and an understanding that trophectoderm biopsy is less invasive than at the cleavage-stage (Scott *et al.*, 2013). Similarly, STR linkage analysis (with or without WGA) was reported as the common testing methodology (85%) (Spinella *et al.*, 2023), with only 8% of all analyses reportedly using WGA and SNP array. Again, however, this is likely misleading as our experience is that Karyomapping has replaced STR linkage analysis for most clinics. Most notably, Spinella *et al.* (2023) report that 13% of embryos had inconclusive results or failed tests (specifically, 7% resulted in failed analyses and 6% had inconclusive diagnoses). In contrast, and concordant with results from a similar study (Poulton *et al.*, 2025), we find here that there was not a single no-result or discrepancy in the ones that were assessed. We stress however that, when potential non-conformities occurred in our own dataset, we extended our analysis window and/or used STR markers; thus, perhaps the comparison of our data and that of Spinella *et al.* (2023) should not be considered as ‘like for like’.

PGT-M is performed to test embryos for a diverse array of inherited genetic diseases and, interestingly, almost half (45.7%) of the disorders tested were unique to individual patients. Although the disorders most frequently tested for in this study were Cystic Fibrosis (n = 834), Fragile X Syndrome (n = 703), and Sickle cell anaemia (n = 462), the second highest relative increase from 2014 to 2021 in testing frequency was for Hereditary breast and ovarian cancer 1 (5.0%). This is also reflected in the disorder groups tested for, where the uptake for PGT-M for cancer-related predispositions and disorders is steadily increasing (15.3%). Such a rising demand may be related to increasing preconception carrier screening (Kraft *et al.*, 2019) or increased knowledge of causative mutations for cancer. Overall, therefore, the uptake for PGT-M is steadily increasing and therefore a concomitant annual rise in testing for novel disorders is noticeable. Elevated genetic screening availability and growing incidence of genetic conditions overall contribute to this trend (Besser *et al.*, 2021), however, there was a temporary decline in 2020, perhaps due to the global COVID-19 pandemic and associated limited access to genetic testing and IVF treatments.

Logically, since the number of genes in the human genome is fixed, eventually most disorders will have been prepared for PGT-M and the number of newly worked-up diseases will decline. Such a trend is reflected to some extent in this study, as a downward slope of −6.9% per year was noted. From Fig. 2C, it is apparent that the decline in novel disorders is itself levelling off at ∼127 diseases new to PGT-M being tested for each year. This is as expected given that the most common disorders will be covered first during assay development, leaving a ‘long tail’ of rare disorders to be worked up as and when new couples present for diagnosis. While this also must eventually converge to zero, with over 6500 single-gene disorders registered in OMIM, it would take more than 40 years at current rates to fully address this diversity. Thus, in practice, there is no indication that we are approaching any ceiling to the number of new unique disorders that can be tested for. Future trends in testing will therefore be governed by regulatory considerations concerning testing eligibility for each disorder, rather than any hypothetical exhaustion of biological possibilities.

What then is the future for PGT-M, either via Karyomapping or successor technologies? While the results of this study suggest that direct mutation analysis for monogenic disorders is no longer strictly necessary (since all the direct mutation analysis that were performed corresponded to the Karyomapping result), retaining direct mutation analysis can be indicated in situations such as germline mosaicism where mutation status and linkage-based results disagree, or where the mutation status of the reference is incorrectly assigned. As technologies yield ever deeper genetic information at ever-lower costs, the practice of PGT-M (and other forms of PGT) will change accordingly. We forecast three key areas of change in the coming years:

First, eventually, the growing density of SNP information gathered by chips of increasing sophistication, and the ever-decreasing cost of whole genome sequencing (WGS) technology, may logically converge at the point where full sequence-level information is gathered for each biopsied embryo. At this point, the distinction between direct mutation testing and linkage-based testing would fall away. The key debate for sequencing-based methods, however, is the depth of coverage that should be obtained, with implications for the cost of the test, for the downstream analysis pipelines, and for the issue of incidental findings of unknown or uncertain significance. Haploblock analysis requires only very low coverage sequencing, while direct mutation testing requires high depth coverage of the targeted locus. It also specifically targets the disease loci of interest. Future advances in sequencing throughput may render the cost issue moot in time, however restricting WGS depth to a lower coverage haploblock analysis approach mitigates the issues of incidental findings that are associated with deeper coverage, even as monogenic, and even polygenic, diagnoses are targeted.

The second area of change is more technical, relating to the nature of the reference sample used to phase the disease-causing mutation with respect to flanking genetic markers. Typically, this is the DNA of a close family member, e.g. an affected child. Such references, however, are not always available. In such cases, it is possible to use a reference within the IVF cycle, such as two or more arrested embryos to set phase-direct mutation testing of the arrested embryos, allowing the phase to be established for the sibling embryos that did not arrest. Where necessary, we applied this approach in this study, thereby demonstrating a further application of Karyomapping over what was first envisioned (Handyside *et al.*, 2010). This too is likely to see expanded use in the future, as preconception carrier screening becomes more popular, allowing couples to identify that they are at risk of transmitting a disorder even in the absence of affected family members serving as a reference. Improvements in sequencing technology will, in turn, likely address this issue too, as long-read sequencing becomes cheaper, allowing the phase of the disease-associated mutation to be identified directly from long-read analysis of the parental DNA.

Finally, while the current study is focused on PGT-M practices, PGT for other indications must also be addressed. Most closely related to PGT-M, the tracing of haploblocks also allows for HLA matching in cases of ‘saviour siblings’ (Delgado *et al.*, 2017). This too may see increased use in the future as this practice becomes more common. More broadly, from its inception, Karyomapping was designed to be able to perform PGT-M, PGT-A (preimplantation genetic testing for aneuploidy), and PGT-SR (preimplantation genetic testing for structural rearrangements) simultaneously, a combination that has since proven to be an accurate tool to estimate the number of usable embryos obtained per cycle (Martel *et al.*, 2024). In linkage-based analyses, monosomy and chromosomal deletions are detected through loss of heterozygosity in the deleted regions in the affected embryos, while trisomy and structural duplications of meiotic (but not of post-zygotic/mitotic) origin are detected by the presence of haploblocks from three different grandparental chromosomes in the duplicated region; uniparental heterodisomy (but not isodisomy) is detected in a similar way. Further analysis of SNP chip raw signal intensity data including the use of Log ratio plots (LRR plots) and B allele frequency plots (BAF plots) allows identification of post-zygotic/mitotic trisomies including mosaic trisomies (Silvestri *et al.*, 2021a). As Karyomapping is not validated for PGT-A in the UK or the USA, we did not use it to detect aneuploidy, nonetheless, we did simultaneously perform regular PGT-A by NGS in 65% of cases (results not shown), indicating that it can be used concurrently with Karyomapping. Whether embryos in which the biopsy result indicates mosaicism should be transferred is an issue of ongoing controversy, although large follow-up studies have shown that unaffected live births are possible after mosaic embryo transfer (Viotti *et al.*, 2023).

While not yet routinely deployed for use in non-human IVF, Karyomapping has been applied to domestic farm animals. PGT for polygenic disease (PGT-P) or commecially important traits to establish genomic estimated breeding values has been combined with PGT-A using Karyomapping and shown to improve live birth rates in cattle by selection of embryos with the highest genetic merit and no aneuploidy (Turner *et al.*, 2019; Silvestri *et al.*, 2021a; Tutt *et al.*, 2021). It has also been used for research into the development of pig embryos (Silvestri *et al.*, 2021b).

Combining linkage-based approaches including Karyomapping with signal-based methods of PGT-A (array comparative genomic hybridization; aCGH) or windowed NGS coverage allows for meiotic and mitotic trisomy to be distinguished, which may prove a useful addition when ranking embryos for selection. This is because, in general terms, meiotic trisomy tends not to lead to live birth, unless a very early trisomy rescue event occurs without UPD. A trisomy detected by NGS but not by Karyomapping would thus be indicative of an error of post-zygotic (mitotic) origin and thus most likely mosaic, regardless of whether all cells in the biopsy were diagnosed aneuploid. Such an approach could therefore, potentially, increase the number of embryos that would be eligible for transfer (Viotti *et al.*, 2023).

## Conclusion

Here, we illustrate the ubiquity of Karyomapping by demonstrating that it has been used in over 14 000 IVF cycles, treating over 9000 couples and identifying over 1000 unique disorders with no reported misdiagnoses. As the approach evolves from SNP-chips to NGS, universal PGT becomes possible, with all variants of PGT (PGT-M, PGT-HLA, PGT-A, PGT-SR, and PGT-P) provided by one single test with high accuracy and reproducibility, and low risk of misdiagnosis. In future, therefore, although the proprietary name ‘Karyomapping’ may well fall into disuse as SNP microarray technology is phased out, its principles will remain as a means of performing universal genetic diagnosis.

## Supplementary Material

deaf198_Supplementary_Data

## Data Availability

The data underlying this article will be shared on reasonable request to the corresponding author.
