# Supplementary material for: Universal preimplantation genetic testing for monogenic disease (Karyomapping): diagnosis of >1000 unique disorders with no detected misdiagnoses
Source: Hum Reprod. 2025 Oct 26;40(12):2440–8. doi: 10.1093/humrep/deaf198 (PMC12675417; doi:10.1093/humrep/deaf198)
Supplement: deaf198_Supplementary_Data [file deaf198_supplementary_data.docx]

Supplementary Table 1: Disorders and Genes/Chromosomes tested for.

| **Disorder** | **Gene/p/q** |
| --- | --- |
| 21-hydroxylase deficient congenital adrenal hyperplasia | CYP21A2 |
| 21-hydroxylase deficient non-classic congenital adrenal hyperplasia | CYP21A2 |
| 3-hydroxyisobutryl-CoA hydrolase deficiency | HIBCH |
| 3-Methylcrotonyl-CoA carboxylase 1 deficiency | MCCC1 |
| 3-Methylcrotonyl-CoA carboxylase 2 deficiency | MCCC2 |
| 3-methylglutaconic aciduria with deafness, encephalopathy, and Leigh-like syndrome | SERAC1 |
| 3-methylglutaconic aciduria-3 | OPA3 |
| 3-methylglutaconic aciduria-7 | CLPB |
| Aarskog-Scott syndrome | FGD1 |
| Achondrogenesis-1B | SLC26A2 |
| Achondroplasia | FGFR3 |
| Achromatopsia 2 | CNGA3 |
| Achromatopsia 3 | CNGB3 |
| Adams-Oliver syndrome 2 | DOCK6 |
| Adams-Oliver syndrome 5 | NOTCH1 |
| Adenosine deaminase deficiency | ADA |
| Adenylosuccinase deficiency | ADSL |
| Adrenoleukodystrophy | ABCD1 |
| Adult Hypophosphatasia | ALPL |
| ADULT syndrome | TP63 |
| Agammaglobulinemia 1 | BTK |
| Aicardi-Goutieres syndrome 1 | TREX1 |
| Aicardi-Goutieres syndrome 2 | RNASEH2B |
| Aicardi-Goutieres syndrome 3 | RNASEH2C |
| Aicardi-Goutieres syndrome 4 | RNASEH2A |
| Aicardi-Goutieres syndrome 5 | SAMHD1 |
| Aicardi-Goutieres syndrome 6 | ADAR |
| Alagille syndrome 1 | JAG1 |
| Alexander disease | GFAP |
| Alkuraya-Kucinskas syndrome | KIAA1109 |
| Allan-Herndon-Dudley syndrome | SLC16A2 |
| Alpha tryptasemia | TPSAB1 |
| Alpha-1-antitrypsin deficiency | SERPINA1 |
| Alpha-Thalassemia | HBA1 |
|  | HBA2 |
| Alpha-thalassemia intellectual disability syndrome | ATRX |
| Alport Syndrome 1 | COL4A5 |
| Alport Syndrome 2 | COL4A4 |
| Alport Syndrome 3 | COL4A3 |
| Alstrom Syndrome | ALMS1 |
| Alzheimer disease-3 | PSEN1 |
| Amelogenesis Imperfecta-3 | FAM83H |
| Amelogenesis imperfecta-4 | DLX3 |
| Amyotrophic lateral sclerosis 1 | SOD1 |
|  | C9orf72 |
| Amyotrophic lateral sclerosis 8 | VAPB |
| Amyotrophic lateral sclerosis 9 | ANG |
| Androgen Insensitivity | AR |
| Angelman syndrome | UBE3A |
| Angioedema 1,2 | SERPING1 |
| Aniridia | PAX6 |
| Ankylosing spondylitis (HLA-B27) | HLA-B27 |
| Anti-D Alloimmunization | RHD |
| Antithrombin III deficiency | SERPINC1 |
| Aortic valve disease 1 | NOTCH1 |
| Apert syndrome | FGFR2 |
| Argininosuccinic aciduria | ASL |
| Arrhythmogenic right ventricular dysplasia 10 | DSG2 |
| Arrhythmogenic right ventricular dysplasia 11 | DSC2 |
| Arrhythmogenic right ventricular dysplasia 8 | DSP |
| Arrhythmogenic right ventricular dysplasia 9 | PKP2 |
| Arterial tortuosity syndrome | SLC2A10 |
| Arthrogryposis, renal dysfunction 1 | VPS33B |
| Arthrogryposis, renal dysfunction 2 | VIPAS39 |
| Asparagine Synthetase Deficiency | ASNS |
| Aspartylglucosaminuria | AGA |
| Ataxia telangiectasia | ATM |
| Auditory neuropathy and optic atrophy | FDXR |
| Autoimmune interstitial lung, joint, and kidney disease | COPA |
| Autoimmune lymphoproliferative syndrome-1A | FAS |
| Autoimmune polyendocrinopathy syndrome-1 | AIRE |
| Axenfeld-Rieger Syndrome-1 | PITX2 |
| Axenfeld-Rieger syndrome-3 | FOXC1 |
| Bardet-Biedl syndrome 1 | BBS1 |
| Bardet-Biedl syndrome 10 | BBS10 |
| Bardet-Biedl syndrome 12 | BBS12 |
| Bardet-Biedl syndrome 6 | MKKS |
| Bardet-Biedl syndrome 7 | BBS7 |
| Bare lymphocyte syndrome-2 | RFX5 |
| Barth syndrome | TAFAZZIN |
| Bartsocas-Papas-1 | RIPK4 |
| Bartter syndrome-1 | SLC12A1 |
| Basal cell nevus syndrome | PTCH1 |
|  | SUFU |
| Becker muscular dystrophy | DMD |
| Beckwith-Wiedemann | CDKN1C |
| Bent bone dysplasia syndrome 2 | LAMA5 |
| Beta Thalassemia - HbE disease | HBB |
| Beta-Thalassemia | HBB |
|  | HBS1L |
|  | HBA2 |
| Bethlem myopathy 1 | COL6A2 |
|  | COL6A1 |
|  | COL6A3 |
| Biotinidase deficiency | BTD |
| Birt-Hogg-Dube syndrome | FLCN |
| Blau syndrome | NOD2 |
| Blepharophimosis, epicanthus inversus, and ptosis | FOXL2 |
| Blood group, ABO system | ABO |
| Blood group, Duffy system | ACKR1 |
| Blood group, MNSs system | GYPA |
| Bloom syndrome | BLM |
| Brain small vessel disease 2 | COL4A2 |
| Branchiooculofacial syndrome | TFAP2A |
| Branchiootorenal syndrome 1 | EYA1 |
| Brugada syndrome 1 | SCN5A |
| Brugada syndrome 3 | CACNA1C |
| Campomelic dysplasia | SOX9 |
| Campomelic dysplasia with autosomal sex reversal | SOX9 |
| Canavan disease | ASPA |
| Capillary, arteriovenous malformation 1 | RASA1 |
| Carbamoyl phosphate synthetase I deficiency | CPS1 |
| Cardiac valvular dysplasia | FLNA |
| Cardiospondylocarpofacial syndrome | MAP3K7 |
| Carney complex-1 | PRKAR1A |
| Carnitine palmitoyltransferase II deficiency | CPT2 |
| Carnitine-acylcarnitine translocase deficiency | SLC25A20 |
| Cartilage-hair hypoplasia | RMRP |
| Cataracts 1 | GJA8 |
| Cataracts 15 | MIP |
| Cataracts 2 | CRYGC |
| Cataracts 6 | EPHA2 |
| Cataracts 9 | CRYAA |
| Catecholaminergic polymorphic ventricular tachycardia 1 | RYR2 |
| Central core disease | RYR1 |
| Cerebellar ataxia, deafness, and narcolepsy | DNMT1 |
| Cerebellar ataxia, mental retardation, and dysequilibrium syndrome 4 | ATP8A2 |
| Cerebellofaciodental syndrome | BRF1 |
| Cerebral arteriopathy with subcortical infarcts and leukoencephalopathy (CADASIL) 1 | NOTCH3 |
| Cerebral cavernous malformation 1 | KRIT1 |
| Cerebral cavernous malformations 2 | CCM2 |
| Cerebral cavernous malformations 3 | PDCD10 |
| Cerebral creatine deficiency syndrome 1 | SLC6A8 |
| Cerebrooculofacioskeletal syndrome 2 | ERCC2 |
| Cerebroretinal microangiopathy with calcifications and cysts | CTC1 |
| Cerebrotendinous Xanthomatosis | CYP27A1 |
| Ceroid lipofuscinosis 3 | CLN3 |
| Ceroid lipofuscinosis 6 | CLN6 |
| Charcot Marie Tooth axonal, 2K | GDAP1 |
| Charcot Marie Tooth B | KARS1 |
| Charcot Marie Tooth Neuropathy 3 | CMTX3 |
| Charcot-Marie-Tooth 1 | GJB1 |
| Charcot-Marie-Tooth 1A | PMP22 |
| Charcot-Marie-Tooth 1E | PMP22 |
| Charcot-Marie-Tooth 4 | AIFM1 |
| Charcot-Marie-Tooth DIE | INF2 |
| Charcot-Marie-Tooth disease-1B | MPZ |
| Charcot-Marie-Tooth-2A | MFN2 |
| Charcot-Marie-Tooth-2P | LRSAM1 |
| CHARGE syndrome | CHD7 |
| Cherubism | SH3BP2 |
| CHIME syndrome | PIGL |
| Chondrodysplasia punctata 1 | ARSL |
| Chondrodysplasia Punctata 2 | EBP |
| Chondrodysplasia-GPAPP | BPNT2 |
| Chondrosarcoma | EXT1 |
|  | EXT2 |
| Choreoacanthocytosis | VPS13A |
| Choroideremia | CHM |
| Chromosome 15q11.2 deletion syndrome | 15q |
|  | DMD |
| Chromosome 15q11-q13 duplication syndrome | 15q |
| Chromosome 15q13.3 microdeletion syndrome | 15q |
| Chromosome 16p11.2 deletion syndrome, 220kb | 16p |
| Chromosome 16p13.3 deletion syndrome | 16p |
| Chromosome 17q21.31 duplication syndrome | 17q21 |
| Chromosome 1q21.1 duplication syndrome | 1q21 |
| Chromosome 1q21.1 deletion syndrome | 1q21 |
| Chromosome 22q11.2 microduplication syndrome | 22q |
| Chromosome 22q11.2 deletion syndrome | 22q |
| Chromosome 2p25.3 deletion syndrome | 2p |
| Chromosome Xp11.22 duplication syndrome | Xp |
| Chromosome Xp22 deletion syndrome | Xp22 |
| Chromosome 17q11.2 duplication syndrome, 1.4Mb | 17q |
| Chromosome 17q12 deletion syndrome | 17q |
| Chromosome 18p deletion syndrome | 18p |
| Chromosome 2p16.3 deletion syndrome | NRXN1 |
|  | 2p |
| Chromosome Xq28 duplication syndrome | Xq |
| Chronic granulomatous disease | CYBB |
| Chronic granulomatous disease 2 | NCF2 |
| Citrullinemia | ASS1 |
| Citrullinemia-2 | SLC25A13 |
| Cleidocranial dysplasia | RUNX2 |
| Cockayne Syndrome-A | ERCC8 |
| Cockayne Syndrome-B | ERCC6 |
| Cohen Syndrome | VPS13B |
| Colorectal adenomas | MUTYH |
| Combined D-2- and L-2-hydroxyglutaric aciduria | SLC25A1 |
| Combined malonic and methylmalonic aciduria | ACSF3 |
| Combined oxidative phosphorylation deficiency 11 | RMND1 |
| Combined oxidative phosphorylation deficiency 12 | EARS2 |
| Combined oxidative phosphorylation deficiency 14 | FARS2 |
| Combined oxidative phosphorylation deficiency 15 | MTFMT |
| Combined oxidative phosphorylation deficiency 16 | MRPL44 |
| Combined oxidative phosphorylation deficiency 20 | VARS2 |
| Combined oxidative phosphorylation deficiency 21 | TARS2 |
| Combined oxidative phosphorylation deficiency 3 | TSFM |
| Combined oxidative phosphorylation deficiency 35 | TRIT1 |
| Combined oxidative phosphorylation deficiency 8 | AARS2 |
| Cone-rod dystrophy 3 | GUCA1A |
| Cone-rod dystrophy 6 | GUCY2D |
| Congenital adrenal hypoplasia | NR0B1 |
| Congenital adrenal insufficiency | CYP11A1 |
| Congenital amegakaryocytic thrombocytopenia | MPL |
| Congenital anomalies of Kidney and Urinary Tract (CAKUT) | CHD1L |
| Congenital bile acid synthesis defect 6 | ACOX2 |
| Congenital cardiac defects | NKX2-5 |
| Congenital central hypoventilation syndrome 1 | PHOX2B |
| Congenital contractural arachnodactyly | FBN2 |
| Congenital contracture syndrome 1 | GLE1 |
| Congenital contracture syndrome 9 | ADGRG6 |
| Congenital disorder of deglycosylation 1 | NGLY1 |
| Congenital disorder of glycosylation-1A | PMM2 |
| Congenital disorder of glycosylation-1B | MPI |
| Congenital disorder of glycosylation-1C | ALG6 |
| Congenital disorder of glycosylation-1D | ALG3 |
| Congenital disorder of glycosylation-1H | ALG8 |
| Congenital disorder of glycosylation-1W | STT3A |
| Congenital disorder of glycosylation-IJ | DPAGT1 |
| Congenital disorders of glycosylation-2I | COG5 |
| Congenital dyserythropoietic anemia 1A | CDAN1 |
| Congenital erythropoietic porphyria | UROS |
| Congenital fibrosis of extraocular muscles 1/3B | KIF21A |
| Congenital fibrosis of extraocular muscles 3A | TUBB3 |
| Congenital glaucoma 3a | CYP1B1 |
| Congenital heart defects 1 | ZIC3 |
| Congenital hydrocephalus 2 | MPDZ |
| Congenital hypofibrinogenemia | FGB |
| Congenital hypomyelinating neuropathy 3 | CNTNAP1 |
| Congenital ichthyosis 1 | TGM1 |
| Congenital Ichthyosis 2 | ALOX12B |
| Congenital Ichthyosis 4A | ABCA12 |
| Congenital Ichthyosis 4B | ABCA12 |
| Congenital insensitivity to pain | NTRK1 |
| Congenital muscular dystrophy 1a | LAMA2 |
| Congenital myasthenia syndrome-1 | CHRNA1 |
| Congenital myasthenia syndrome-2 | CHRNB1 |
| Congenital myasthenic syndrome 10 | DOK7 |
| Congenital myasthenic syndrome 11 | RAPSN |
| Congenital myasthenic syndrome 20 | SLC5A7 |
| Congenital myasthenic syndrome 22 | PREPL |
| Congenital myasthenic syndrome 4 | CHRNE |
| Congenital myasthenic syndrome 6 | CHAT |
| Congenital Myopathy | STAC3 |
| Congenital neutropenia 3 | HAX1 |
| Congenital nystagmus 1 | FRMD7 |
| Corpus callosum agenesis | SLC12A6 |
|  | IGBP1 |
| Cowden syndrome 1 | PTEN |
| Cranioectodermal dysplasia 2 | WDR35 |
| Craniofrontonasal dysplasia | EFNB1 |
| Craniosynostosis with radiohumeral fusions and other skeletal and craniofacial anomalies | CYP26B1 |
| Craniosynostosis, nonspecific | FGFR2 |
| CRASH syndrome | L1CAM |
| Creutzfeldt-Jakob disease | PRNP |
| Crouzon syndrome | FGFR2 |
| Currarino syndrome | MNX1 |
| Cutis laxa 1a | FBLN5 |
| Cyclic Ichthyosis with erpidermolytic hyperkeratosis | KRT1 |
| Cyclic neutropenia | ELANE |
| Cystic Fibrosis | CFTR |
| Danon disease | LAMP2 |
| D-bifunctional protein deficiency | HSD17B4 |
| Deafness 1, with or without thrombocytopenia | DIAPH1 |
| Deafness 10 | EYA4 |
| Deafness 15 | POU4F3 |
| Deafness 16 | STRC |
| Deafness 1a | GJB2 |
| Deafness 1b | GJB6 |
| Deafness 22 | MYO6 |
| Deafness 29 | CLDN14 |
| Deafness 3 | MYO15A |
| Deafness 4 | SLC26A4 |
| Deafness 4, with enlarged vestibular aqueduct | SLC26A4 |
| Deafness 6 | COL4A6 |
| Deafness 84B | OTOGL |
| Deafness 86 | TBC1D24 |
| Deafness, dystonia, and cerebral hypomyelination | BCAP31 |
| Dehydrated hereditary stomatocytosis | PIEZO1 |
| Dent disease 2 | OCRL |
| Dentatorubral pallidoluysian atrophy | ATN1 |
| Dentici-Novelli neurodevelopmental syndrome | ZNF526 |
| Dentinogenesis imperfecta | DSPP |
| Desbuquois dysplasia 1 | CANT1 |
| Desmosterolosis | DHCR24 |
| Developmental and epileptic encephalopathy 1 | ARX |
| Developmental and epileptic encephalopathy 18 | SZT2 |
| Developmental and epileptic encephalopathy 28 | WWOX |
| Developmental and epileptic encephalopathy 29 | AARS1 |
| Developmental and epileptic encephalopathy 35 | ITPA |
| Developmental and epileptic encephalopathy 37 | FRRS1L |
| Developmental and epileptic encephalopathy 4 | STXBP1 |
| Developmental and epileptic encephalopathy 44 | UBA5 |
| Developmental and epileptic encephalopathy 7 | KCNQ2 |
| Developmental and epileptic encephalopathy 8 | ARHGEF9 |
| Developmental and epileptic encephalopathy 95 | PIGS |
| Diabetes mellitus, insulin-resistant | INSR |
| Diamond-Blackfan anemia 1 | RPS19 |
| Diamond-Blackfan anemia 7 | RPL11 |
| Diarrhea 2, with microvillus atrophy | MYO5B |
| Diastrophic dysplasia | SLC26A2 |
| DiGeorge syndrome | 22q |
|  | TBX1 |
| Dihydrolipoamide dehydrogenase deficiency | DLD |
| Dihydropyrimidine dehydrogenase deficiency | DPYD |
| Dilated cardiomyopathy | DSP |
| Dilated cardiomyopathy 1A | LMNA |
| Dilated cardiomyopathy 1DD | RBM20 |
| Dilated cardiomyopathy 1E | SCN5A |
| Dilated cardiomyopathy 1G | TTN |
| Dilated cardiomyopathy 1P | PLN |
| Dilated cardiomyopathy 1S | MYH7 |
| Dilated cardiomyopathy 1W | VCL |
| Dilated cardiomyopathy 1Y | TPM1 |
| Dilated cardiomyopathy 3D | DMD |
| Distal arthrogryposis-2B1 | TNNI2 |
| Distal arthrogryposis-2B2 | TNNT3 |
| Distal Arthrogryposis-5 | PIEZO2 |
| Dravet syndrome | SCN1A |
| Duane-radial ray syndrome | SALL4 |
| Duchenne muscular dystrophy | DMD |
| Dyskeratosis congenita 1 | DKC1 |
|  | TERC |
| Dyskeratosis congenita 2 | TERT |
| Dyskeratosis congenita 3 | TINF2 |
| Dyskeratosis congenita 4 | RTEL1 |
| Dyskeratosis congenita 5 | RTEL1 |
| Dyssegmental dysplasia-Silverman-Handmaker | HSPG2 |
| Dystonia-Parkinsonism | TAF1 |
| Dystrophic epidermolysis bullosa (AD) | COL7A1 |
| Ectodermal dysplasia 10A | EDAR |
| Ectopia Lentis 1 | FBN1 |
| Ectrodactyly, ectodermal dysplasia, cleft lip/palate syndrome 3 | TP63 |
| Ehlers-Danlos syndrome 1 | COL5A1 |
|  | TNXB |
| Ehlers-Danlos syndrome 1 (peridontal) | C1R |
| Ehlers-Danlos syndrome 1, kyphoscoliotic type | PLOD1 |
| Ehlers-Danlos syndrome 2 | B3GALT6 |
| Ehlers-Danlos syndrome-vascular | COL3A1 |
| Ellis-Van Creveld Syndrome | EVC2 |
|  | EVC |
| Emery-Dreifuss muscular dystrophy 1 | EMD |
| Emery-Dreifuss muscular dystrophy 2 | LMNA |
| Encephalopathy 1 | DNM1L |
| Epidermolysis bullosa dystrophica (AR) | COL7A1 |
| Epidermolytic Hyperkeratosis | KRT10 |
| Epidermolytic palmoplantar keratoderma | KRT9 |
| Epilepsy-aphasia syndrome | GRIN2A |
| Episodic ataxia-2 | CACNA1A |
| Erythrokeratodermia variabilis et progressiva 1 | GJB3 |
| Escobar Syndrome | CHRNG |
| Exostoses-1 | EXT1 |
| Exostoses-2 | EXT2 |
| Exudative vitreoretinopathy 1 | FZD4 |
| Exudative vitreoretinopathy 4 | LRP5 |
| Exudative vitreoretinopathy 5 | TSPAN12 |
| Fabry disease | GLA |
| Factor V Deficiency | F5 |
| Factor XI deficiency | F11 |
| Familial adenomatous polyposis | APC |
| Familial dysautonomia | ELP1 |
| Familial focal Epilepsy 1 | DEPDC5 |
| Familial hypercholesterolemia 1 | LDLR |
| Familial hyperinsulinemic hypoglycemia 1 | ABCC8 |
| Familial mediterranean fever | MEFV |
| Familial osteochondritis dissecans | ACAN |
| Familial platelet disorder | RUNX1 |
| Familial thoracic aortic aneurysm 6 | ACTA2 |
| Fanconi anemia A | FANCA |
| Fanconi anemia B | FANCB |
| Fanconi anemia C | FANCC |
| Fanconi anemia F | FANCF |
| Fanconi anemia G | FANCG |
| Fanconi anemia J | BRIP1 |
| Fanconi-Bickel syndrome | SLC2A2 |
| Farber lipogranulomatosis | ASAH1 |
| Fascioscapulohumeral muscular dystrophy 1 | DUX4 |
| Fascioscapulohumeral muscular dystrophy 2 | SMCHD1 |
| Fatal Insomnia | PRNP |
| Febrile seizures 2 | HCN2 |
| Febrile seizures 3a | SCN1A |
| Feingold syndrome 1 | MYCN |
| Fetal akinesia deformation | NMNAT2 |
| Fibrodysplasia ossificans progressiva | ACVR1 |
| Focal dermal hypoplasia | PORCN |
| Focal segmental glomerulosclerosis 1 | ACTN4 |
| Foveal hypoplasia 2 | SLC38A8 |
| Fragile X syndrome | FMR1 |
| Fraser syndrome 1 | FRAS1 |
| Fraser syndrome 2 | FREM2 |
| Frontotemporal dementia | GRN |
|  | MAPT |
|  | VCP |
| Frontotemporal dementia/amyotrophic lateral sclerosis 1 | C9orf72 |
| Fuhrmann syndrome | WNT7A |
| Fumarase Deficiency | FH |
| Galactosemia | GALT |
| Galloway-Mowat syndrome 3 | OSGEP |
| Gastrointestinal defects and immunodeficiency syndrome | TTC7A |
| Gaucher disease-1 | GBA |
| Generalised severe Epidermolysis bullosa simplex 1A | KRT14 |
| Generalized arterial calcification of infancy 1 | ENPP1 |
| Generalized Epilepsy with febrile seizures 2 | SCN1A |
| Gerstmann-Straussler disease | PRNP |
| Glomerulopathy with fibronectin deposits 2, Spondylometaphyseal dysplasia | FN1 |
| Glomerulosclerosis 5 | INF2 |
| Glutaric acidemia 1 | GCDH |
| Glutaric acidemia 2A | ETFA |
| Glutaric acidemia 2C | ETFDH |
| Glycine encephalopathy | GLDC |
|  | ATM |
| Glycogen storage disease 1a | G6PC1 |
| Glycogen storage disease 2 | GAA |
| Glycogen storage disease 3 | AGL |
| Glycogen storage disease 4 | GBE1 |
| Glycogen storage disease 5 | PYGM |
| Glycogen storage disease-9a | PHKA2 |
| GM1-gangliosidosis | GLB1 |
| GM2-gangliosidosis AB | GM2A |
| Goldberg-Shprintzen megacolon syndrome | KIFBP |
| GRACILE syndrome | BCS1L |
| Greig cephalopolysyndactyly syndrome | GLI3 |
| Hailey-Hailey disease | ATP2C1 |
| Harel-Yoon syndrome | ATAD3A |
| HARP Syndrome | PANK2 |
| Hay-Wells Syndrome | TP63 |
| Hearing loss 20/26 | ACTG1 |
| Hearing loss 6/14/38 | WFS1 |
| Hearing loss 8/10 | TMPRSS3 |
| Hemochromatosis | HFE |
| Hemoglobin E beta-thalassemia | HBB |
| Hemolytic Amemia | G6PD |
| Hemophagocytic lymphohistiocytosis 2 | PRF1 |
| Hemophagocytic lymphohistiocytosis 3 | UNC13D |
| Hemophagocytic lymphohistiocytosis 5 | STXBP2 |
| Hemophilia A | F8 |
| Hemophilia B | F9 |
| Hennekam lymphangiectasia-lymphedema syndrome 1 | CCBE1 |
| Hereditary breast-ovarian cancer 1 | BRCA1 |
| Hereditary breast-ovarian cancer 2 | BRCA2 |
| Hereditary diffuse gastric cancer | CDH1 |
| Hereditary Fructose Intolerance | ALDOB |
| Hereditary hemorrhagic telangiectasia-1 | ENG |
| Hereditary hemorrhagic telangiectasia-2 | ACVRL1 |
| HEX A Pseudodeficiency | HEXA |
| Hidrotic Ectodermal Dysplasia 2 | GJB6 |
| Hirschsprung disease 1 | RET |
| Histiocytosis-lymphadenopathy plus syndrome | SLC29A3 |
| Holoprosencephaly 2 | SIX3 |
| Holoprosencephaly 3 | SHH |
| Holoprosencephaly 5 | ZIC2 |
| Holt-Oram syndrome | TBX5 |
| Homocystinuria | CBS |
|  | MTHFR |
| Huntington Disease | HTT |
| Hurler Syndrome | IDUA |
| Hutchinson-Gilford progeria | LMNA |
| Hyaline fibromatosis syndrome | ANTXR2 |
| Hydrocephalus | L1CAM |
| Hydrolethalus syndrome | HYLS1 |
| Hydrops, lactic acidosis, and sideroblastic anemia | LARS2 |
| Hyper IgE Recurrent Infection Syndrome 2 | DOCK8 |
|  | STAT3 |
| Hyperekplexia 1 | GLRA1 |
| Hyperekplexia 3 | SLC6A5 |
| Hyperglycinemia, lactic acidosis, seizures | LIAS |
| Hyper-IgM immunodeficiency | CD40LG |
| Hyperinsulinemic hypoglycemia-2 | KCNJ11 |
| Hyperkalemic periodic paralysis-2 | SCN4A |
| Hyperoxaluria-1 | AGXT |
| Hyperphenylalaninemia A | PTS |
| Hyperphosphatasia syndrome with mental retardation 4 | PGAP3 |
| Hypertension, brachydactyly syndrome | PDE3A |
| Hypertrichotic osteochondrodysplasia | ABCC9 |
| Hypertrophic cardiomyopathy 1 | MYH7 |
| Hypertrophic cardiomyopathy 10 | MYL2 |
| Hypertrophic cardiomyopathy 11 | ACTC1 |
| Hypertrophic cardiomyopathy 2 | TNNT2 |
| Hypertrophic cardiomyopathy 26 | FLNC |
| Hypertrophic cardiomyopathy 3 | TPM1 |
| Hypertrophic cardiomyopathy 4 | MYBPC3 |
| Hypertrophic cardiomyopathy 7 | TNNI3 |
| Hypertrophic cardiomyopathy 8 | MYL3 |
| Hypogonadotropic hypogonadism 1 | ANOS1 |
| Hypogonadotropic hypogonadism 2 | FGFR1 |
| Hypogonadotropic hypogonadism 3 | PROKR2 |
| Hypogonadotropic hypogonadism 6 | FGF8 |
| Hypohidrotic ectodermal dysplasia 1 | EDA |
| Hypokalemic Periodic Paralysis-1 | CACNA1S |
| Hypoparathyroidism-retardation-dysmorphism syndrome | TBCE |
| Hypophosphatemic rickets | PHEX |
| Hypoplastic left heart Syndrome | MYH6 |
|  | NOTCH1 |
| Hypotonia with psychomotor development disorders 2 | UNC80 |
| Hypotonia, ataxia, and developmental delay syndrome | EBF3 |
| Ichthyosis | STS |
| IMAGE syndrome | CDKN1C |
| Immunodeficiency 14 | PIK3CD |
| Immunodeficiency 18 | CD3E |
| Immunodeficiency 21 | GATA2 |
| Immunodeficiency 27A | IFNGR1 |
| Immunodeficiency 64 | RASGRP1 |
| Immunodeficiency 67 | IRAK4 |
| Immunodeficiency 9 | ORAI1 |
| Immunodysregulatory polyendocrinopahy, enteropathy | FOXP3 |
| Incontinentia pigmenti | IKBKG |
| Infantile cerebellar retinal degeneration | ACO2 |
| Infantile convulsions | PRRT2 |
| Infantile hypercalcemia 2 | SLC34A1 |
| Infantile Hypophosphatasia | ALPL |
| Infantile Hypotonia 1 | NALCN |
| Infantile liver failure syndrome 1 | LARS1 |
| Infantile myofibromatosis 1 | PDGFRB |
| Infantile Nephronophthisis 2 | INVS |
| Infantile neuroaxonal dystrophy 1 | PLA2G6 |
| Infantile onset ascending spastic paralysis | ALS2 |
| Infantile seizures 2 | PRRT2 |
| Intellectual development disorder and microcephaly | CASK |
| Intellectual developmental disorder 1 | MBD5 |
| Intellectual developmental disorder 109 | AFF2 |
| Intellectual developmental disorder 12 | ST3GAL3 |
| Intellectual developmental disorder 14 | UPF3B |
| Intellectual developmental disorder 29 | ARX |
| Intellectual developmental disorder 3 | HCFC1 |
| Intellectual developmental disorder 58 | TSPAN7 |
| Intellectual developmental disorder 9 | FTSJ1 |
| Intellectual developmental disorder 99 | USP9X |
| Intellectual developmental disorder with cardiac defects and dysmorphic facies | TMEM94 |
| Intellectual developmental disorder with dysmorphic facies, speech delay, and T-cell abnormalities | BCL11B |
| Intellectual developmental disorder with paroxysmal dyskinesia or seizures | PDE2A |
| Intellectual developmental disorder, Claes-Jensen type | KDM5C |
| Intellectual developmental disorder, Lubs | MECP2 |
| Intellectual developmental disorder-Billuart | OPHN1 |
| Intellectual developmental disorder-Christianson | SLC9A6 |
| Intellectual developmental disorder-Nascimento | UBE2A |
| Intellectual developmental disorder-Snijders Blok | DDX3X |
| Intellectual developmental disorder-Turner | HUWE1 |
| Intellectual disability-hypotonic facies syndrome | ATRX |
| Interleukin 1 receptor antagonist deficiency | IL1RN |
| Intermediate junctional Epidermolysis bullosa 1A | LAMB3 |
| Intestinal pseudo-obstruction | FLNA |
| Iron-refractory iron deficiency anemia | TMPRSS6 |
| Isovaleric acidemia | IVD |
| Jervell and Lange-Nielsen syndrome | KCNQ1 |
| Joubert Syndrome 12 | KIF7 |
| Joubert Syndrome 18 | TCTN3 |
| Joubert syndrome 2 | TMEM216 |
| Joubert syndrome 21 | CSPP1 |
| Joubert syndrome 3 | AHI1 |
| Joubert syndrome 38 | KIAA0753 |
| Joubert syndrome 4 | NPHP1 |
| Joubert syndrome 5 | CEP290 |
| Joubert syndrome 6 | TMEM67 |
| Joubert syndrome 9 | CC2D2A |
| Junctional Epidermolysis Bullosa 2 | LAMA3 |
| Junctional Epidermolysis Bullosa 3 | LAMC2 |
| Junctional Epidermolysis bullosa 5B | ITGB4 |
| Juvenile intestinal polyposis | BMPR1A |
| Kabuki syndrome 1 | KMT2D |
| Kell blood group antigen | KEL |
| Knobloch Syndrome-1 | COL18A1 |
| Krabbe disease | GALC |
| L-2-hydroxyglutaric aciduria | L2HGDH |
| LADD syndrome | FGFR2 |
|  | FGF10 |
| Laing distal myopathy | MYH7 |
| L-amino acid decarboxylase deficiency | DDC |
| Larsen Syndrome | FLNB |
| Laryngoonychocutaneous syndrome | LAMA3 |
| LCHAD deficiency | HADHA |
| Leber congenital amaurosis 1 | GUCY2D |
| Leber congenital amaurosis 10 | CEP290 |
| Leber congenital amaurosis 2 | RPE65 |
| Leber congenital amaurosis 4 | AIPL1 |
| Leber congenital amaurosis 8 | CRB1 |
| Leber congenital amaurosis 9 | NMNAT1 |
| Leiomyomatosis and renal cell cancer | FH |
| LEOPARD syndrome 1 | PTPN11 |
| Leptin receptor deficiency | LEPR |
| Leri-Weill dyschondrosteosis | SHOX |
| Lesch-Nyhan syndrome | HPRT1 |
| Lesch-Nyhan syndrome | HPRT1 |
| Leukodystrophy 7/Wiedeman-Rautenstrauch syndrome | POLR3A |
| Leukodystrophy-13 | HIKESHI |
| Leukoencephalopathy with brain stem and spinal cord involvement and lactate elevation | DARS2 |
| Leukoencephalopathy with vanishing white matter | EIF2B2 |
| Li-Fraumeni syndrome | TP53 |
| Li-Fraumeni Syndrome 2 | CHEK2 |
| Limb-girdle muscular dystrophy 1 | CAPN3 |
| Limb-girdle muscular dystrophy 2 | DYSF |
| Limb-girdle muscular dystrophy 4 | SGCB |
| Limb-girdle muscular dystrophy 5 | SGCG |
| Lipoid adrenal hyperplasia | STAR |
| Lipoyltransferase 1 Deficiency | LIPT1 |
| Lissencephaly 1 | DCX |
| Lissencephaly 2 | ARX |
| Localised Epidermolysis Bullosa simplex 2C | KRT5 |
| Loeys-Dietz syndrome 1 | TGFBR1 |
| Loeys-Dietz syndrome 2 | TGFBR2 |
| Loeys-Dietz syndrome 3 | SMAD3 |
| Loeys-Dietz syndrome 4 | TGFB2 |
| Loeys-Dietz syndrome 5 | TGFB3 |
| Long QT syndrome 1 | KCNQ1 |
| Long QT syndrome 2 | KCNH2 |
| Long QT syndrome 3 | SCN5A |
| Long QT Syndrome 8 | CACNA1C |
| Lowe syndrome | OCRL |
| Luscan-Lumish syndrome | SETD2 |
| Lymphatic malformation 1 | FLT4 |
| Lymphatic malformation 6 | PIEZO1 |
| Lymphedema-distichiasis syndrome | FOXC2 |
| Lymphoproliferative syndrome 2 | XIAP |
| Lynch Syndrome-1 | MSH2 |
| Lynch Syndrome-2 | MLH1 |
| Lynch Syndrome-4 | PMS2 |
| Lynch Syndrome-5 | MSH6 |
| Lynch Syndrome-8 | EPCAM |
| Lysosomal Acid Lipase deficiency | LIPA |
| Machado-Joseph disease | ATXN3 |
| Macular dystrophy, vitelliform 2 | BEST1 |
| Malan Syndrome | NFIX |
| Maple syrup urine disease-1a | BCKDHA |
| Maple syrup urine disease-1b | BCKDHB |
| Maple syrup urine disease-2 | DBT |
| Marfan syndrome | FBN1 |
| Martsolf syndrome 1 | RAB3GAP2 |
| Maturity-onset diabetes of the young (MODY)-1 | HNF4A |
| MCLMR syndrome | KIF11 |
| Meckel syndrome 1 | MKS1 |
| Meckel syndrome 3 | TMEM67 |
| Meckel syndrome 6 | CC2D2A |
| Meckel Syndrome 7 | NPHP3 |
| Meckel syndrome 9 | B9D1 |
| Medium-chain acyl-CoA dehydrogenase deficiency | ACADM |
| Megacystis-microcolon-intestinal hypoperistalsis syndrome (MMIHS) 4 | MYL9 |
| Melanoma-pancreatic cancer syndrome | CDKN2A |
| Meleda disease | SLURP1 |
| Menkes disease | ATP7A |
| Mental retardation 37 | ANK3 |
| Metachromatic Leukodystrophy | ARSA |
| Metaphyseal chondrodysplasia, Schmid type | COL10A1 |
| Methylmalonic aciduria mut(0)-type | MMUT |
| Methylmalonic aciduria cb1B-type | MMAB |
| Methylmalonic aciduria and homocystinuria | MMACHC |
| Microcephalic osteodysplastic primordial dwarfism-1 | RNU4ATAC |
| Microcephalic osteodysplastic primordial dwarfism-2 | PCNT |
| Microcephaly and chorioretinopathy 1 | TUBGCP6 |
| Microcephaly, seizures and developmental delay | PNKP |
| Microcephaly-capillary malformation syndrome | STAMBP |
| Midface hypoplasia, hearing impairment, elliptocytosis, and nephrocalcinosis | AMMECR1 |
| Mitochondrial complex 1 deficiency-16 | NDUFAF5 |
| Mitochondrial complex 1 deficiency-20 | ACAD9 |
| Mitochondrial complex 1 deficiency-21 | NUBPL |
| Mitochondrial complex 1 deficiency-28 | NDUFA13 |
| Mitochondrial complex 1 deficiency-5 | NDUFS1 |
| Mitochondrial complex 1 deficiency-6 | NDUFS2 |
| Mitochondrial complex 1 deficiency-7 | NDUFV2 |
| Mitochondrial complex 3 deficiency-1 | BCS1L |
| Mitochondrial complex 4 deficiency-1 | SURF1 |
| Mitochondrial complex II deficiency-1 | SDHA |
| Mitochondrial complex IV deficiency-5 | LRPPRC |
| Mitochondrial complex IV deficiency-6 | COX15 |
| Mitochondrial DNA depletion syndrome 13 | FBXL4 |
| Mitochondrial DNA depletion syndrome 2 | TK2 |
| Mitochondrial DNA depletion syndrome 3 | DGUOK |
| Mitochondrial DNA depletion syndrome 4a | POLG |
| Mitochondrial DNA depletion syndrome 4b | POLG |
| Mitochondrial DNA depletion syndrome 5 | SUCLA2 |
| Mitochondrial DNA depletion syndrome 6 | MPV17 |
| Mitochondrial DNA depletion syndrome 8 | RRM2B |
| Mitochondrial DNA depletion syndrome 9 | SUCLG1 |
| Mitochondrial induced deafness | TRMU |
| Mitochondrial short-chain enoyl-CoA hydratase 1 deficiency | ECHS1 |
| Mitochondrial trifunctional protein deficiency | HADHB |
| Mixed polyposis syndrome | GREM1 |
| MODY-4 | PDX1 |
| Mohr-Tranebjaerg Syndrome | TIMM8A |
| Monilethrix | KRT86 |
|  | KRT81 |
| Muckle-Wells syndrome | NLRP3 |
| Mucolipidosis II alpha/beta | GNPTAB |
| Mucolipidosis III alpha/beta | GNPTAB |
| Mucolipidosis III gamma | GNPTG |
| Mucolipidosis IV | MCOLN1 |
| Mucopolysaccharidosis II | IDS |
| Mucopolysaccharidosis IVA | GALNS |
| Mucopolysaccharidosis VII | GUSB |
| Mucopolysaccharidosis IIIA (Sanfilippo A) | SGSH |
| Mucopolysaccharidosis IIIB (Sanfilippo B) | NAGLU |
| Mucopolysaccharidosis IIIC (Sanfilippo C) | HGSNAT |
| Mucopolysaccharidosis VI (Maroteaux-Lamy) | ARSB |
| Muenke syndrome | FGFR3 |
| Mulibrey nanism | TRIM37 |
| Multiple congenital anomalies-hypotonia-seizures syndrome 1 | PIGN |
| Multiple congenital anomalies-hypotonia-seizures syndrome 2 | PIGA |
| Multiple Endocrine Neoplasia 1 | MEN1 |
| Multiple endocrine neoplasia 2A | RET |
| Multiple endocrine neoplasia 2B | RET |
| Multiple epiphyseal dysplasia 1 | COMP |
| Multiple epiphyseal dysplasia 2 | COL9A2 |
| Multiple epiphyseal dysplasia 5 | MATN3 |
| Multiple epiphyseal dysplasia 7 | CANT1 |
| Multiple epiphyseal dysplasia with early onset Diabetes Mellitus | EIF2AK3 |
| Multiple mitochondrial dysfunctions syndrome 4­ | ISCA2 |
| Multiple pterygium syndrome, lethal type | CHRNG |
| Multiple sulfatase deficiency | SUMF1 |
| Multiple synostosis syndrome 1/Tarsal-carpal coalition syndrome | NOG |
| Muscular dystrophy 10 | TTN |
| Muscular dystrophy-dystroglycanopathy 11 | B3GALNT2 |
| Muscular dystrophy-dystroglycanopathy 4 | FKTN |
| Muscular dystrophy-dystroglycanopathy-14 | GMPPB |
| Muscular dystrophy-dystroglycanopathy-1A | POMT1 |
| Muscular dystrophy-dystroglycanopathy-1C | POMT1 |
| Muscular dystrophy-dystroglycanopathy-2 | POMT2 |
| Muscular dystrophy-dystroglycanopathy-3A | POMGNT1 |
| Muscular dystrophy-dystroglycanopathy-4(a) | FKTN |
| Muscular dystrophy-dystroglycanopathy-5 | FKRP |
| Myoclonic dystonia-11 | SGCE |
| Myofibrillar myopathy 1 | DES |
| Myofibrillar myopathy 4 | LDB3 |
| Myotonia congenita | CLCN1 |
| Myotonic dystrophy 1 | DMPK |
| Myotonic dystrophy 2 | CNBP |
| Myotubular myopathy | MTM1 |
| Nail-patella syndrome | LMX1B |
| Nemaline myopathy 2 | NEB |
| Nemaline Myopathy 3 | ACTA1 |
| Nemaline myopathy 8 | KLHL40 |
| Nemaline myopathy-10 | LMOD3 |
| Neonatal alloimmune thrombocytopenia | ITGB3 |
| Nephrogenic diabetes insipidus 1 | AVPR2 |
| Nephropathic cystinosis | CTNS |
| Nephropathy | CFHR5 |
| Nephrotic syndrome-1 | NPHS1 |
| Nephrotic syndrome-2 | NPHS2 |
| Netherton syndrome | SPINK5 |
| Neurodegeneration with brain iron accumulation 2B | PLA2G6 |
| Neurodegeneration with brain iron accumulation 5 | WDR45 |
| Neurodegeneration with choreoathetoid movements | IREB2 |
| Neurodevelopmental disorder with hearing loss, seizures, and brain abnormalities | SPATA5 |
| Neurodevelopmental disorder with microcephaly, seizures, and cortical atrophy | VARS1 |
| Neurofibromatosis-1 | NF1 |
| Neurofibromatosis-2 | NF2 |
| Neuronal ceroid lipofuscinosis 1 | PPT1 |
| Neuronal ceroid lipofuscinosis 2 | TPP1 |
| Neuronopathy-2a | HSPB8 |
| Neuronopathy-6 | IGHMBP2 |
| Neuropathy-6 | DST |
| Niemann-Pick disease-A | SMPD1 |
| Niemann-Pick disease-B | SMPD1 |
| Niemann-Pick disease-C1 | NPC1 |
|  | SMPD1 |
| Niemann-Pick disease-C2 | NPC2 |
| Nijmegen breakage syndrome | NBN |
| Nonaka myopathy | GNE |
| Non-epidermolytic palmoplantar keratoderma | KRT1 |
| Non-syndromic nail disorder 1 | FZD6 |
| Noonan syndrome 1 | PTPN11 |
| Noonan syndrome 10 | LZTR1 |
| Noonan syndrome 2 | LZTR1 |
| Noonan syndrome 4 | SOS1 |
| Noonan syndrome 5 | RAF1 |
| Noonan Syndrome 6 | NRAS |
| Noonan syndrome 8 | RIT1 |
| Norrie disease | NDP |
| Ocular Albinism-1 | GPR143 |
| Oculocutaneous albinism-1A | TYR |
| Oculocutaneous Albinism-2 | OCA2 |
| Oculocutaneous albinism-4 | SLC45A2 |
| Oculodentodigital dysplasia | GJA1 |
| Oculopharyngeal muscular dystrophy | PABPN1 |
| Odontoonychodermal dysplasia | WNT10A |
| Omenn syndrome | RAG2 |
| Opitz GBBB syndrome-1 | MID1 |
| Opitz-Kaveggia syndrome | MED12 |
| Optic atrophy 1 | OPA1 |
| Ornithine transcarbamylase deficiency | OTC |
| Osseous heteroplasia | GNAS |
| Osteogenesis imperfecta 1 | COL1A1 |
| Osteogenesis imperfecta 3 | COL1A1 |
| Osteogenesis imperfecta 4 | COL1A2 |
| Osteogenesis imperfecta XX | MESD |
| Osteogenesis imperfecta-19 | MBTPS2 |
| Osteogenesis imperfecta-8 | P3H1 |
| Osteogenesis imperfecta-9 | PPIB |
| Osteopetrosis 1 | TCIRG1 |
| Osteopetrosis 2 | CLCN7 |
| Otopalatodigital syndrome-2 | FLNA |
| Pachyonychia congenita 2 | KRT17 |
| Pachyonychia congenita 3 | KRT6A |
| Pancreatitis | PRSS1 |
|  | SPINK1 |
| Papillorenal syndrome | PAX2 |
| Paragangliomas 1 | SDHD |
| Paragangliomas 4 | SDHB |
| Paramyotonia congenita | SCN4A |
| Partial lipodystrophy-2 | LMNA |
| Patterned macular dystrophy 1 | PRPH2 |
| Pelizaeus-Merzbacher disease | PLP1 |
|  | Xq22 |
| Pendred syndrome | SLC26A4 |
| Periodic Fever syndrome | TNFRSF1A |
| Periventricular heterotopia 1 | FLNA |
| Peroxisomal acyl-CoA oxidase deficiency | ACOX1 |
| Peroxisome biogenesis disorder 11A (Zellweger) | PEX13 |
| Peroxisome biogenesis disorder 5A (Zellweger) | PEX2 |
| Peroxisome biogenesis disorder-1A | PEX1 |
| Peroxisome biogenesis disorder-3A | PEX12 |
| Peroxisome biogenesis disorder-4A | PEX6 |
| Peroxisome biogenesis disorder-6A | PEX10 |
| Peroxisome biogenesis disorder-7A | PEX26 |
| Peutz-Jeghers syndrome | STK11 |
| Phenylketonuria | PAH |
| Pheochromocytoma | SDHB |
|  | RET |
| Pierson syndrome | LAMB2 |
| Pitt-Hopkins-like syndrome 2 | NRXN1 |
| Pleuropulmonary blastoma | DICER1 |
| Poikiloderma, Hereditary Fibrosing, Myopathy, and Pulmonary Fibrosis | FAM111B |
| Polycystic kidney disease 1 | PKD1 |
| Polycystic kidney disease 2 | PKD2 |
| Polycystic kidney disease 4 | PKHD1 |
| Pontocerebellar hypoplasia 7 | TOE1 |
| Pontocerebellar hypoplasia-1B | EXOSC3 |
| Pontocerebellar hypoplasia-2A | TSEN54 |
| Pontocerebellar hypoplasia-2D | SEPSECS |
| Pontocerebellar hypoplasia-4 | TSEN54 |
| Pontocerebellar hypoplasia-6 | RARS2 |
| Popliteal pterygium syndrome 1 | IRF6 |
| Posterior Amelia | TBX4 |
| Precocious puberty | LHCGR |
| Primary amyloidosis 1 | OSMR |
| Primary carnitine deficiency | SLC22A5 |
| Primary ciliary dyskinesia 11 | RSPH4A |
| Primary ciliary dyskinesia 18 | DNAAF5 |
| Primary ciliary dyskinesia 3 | DNAH5 |
| Primary ciliary dyskinesia 37 | DNAH1 |
| Primary ciliary dyskinesia 7 | DNAH11 |
| Primary ciliary dyskinesia 9 | DNAI2 |
| Primary coenzyme Q10 deficiency 1 | COQ9 |
|  | COQ2 |
|  | COQ4 |
| Primary hyperoxaluria-2 | GRHPR |
| Primary hyperoxaluria-3 | HOGA1 |
| Primary microcephaly 2 | WDR62 |
| Primary microcephaly 5 | ASPM |
| Primary microcephaly 7 | STIL |
| Primary Microcephaly 8 | CEP135 |
| Primary pulmonary hypertension-1 | BMPR2 |
| Progressive Encephalopathy | TBCD |
| Progressive external ophthalmoplegia 1 | POLG |
| Progressive Familial Intrahepatic Cholestasis Type 2 | ABCB11 |
| Progressive myoclonic epilepsy 3 | KCTD7 |
| Progressive Myoclonic Epilepsy 6 | GOSR2 |
| Prolidase deficiency | PEPD |
| Proliferative vasculopathy and hydranencephaly-hydrocephaly syndrome | FLVCR2 |
| Propionicacidemia | PCCA |
|  | PCCB |
| Pseudoachondroplasia | COMP |
| Pseudohermaphroditism | HSD17B3 |
| Pseudohypoparathyroidism-1a | GNAS |
| Pseudovaginal perineoscrotal hypospadias | SRD5A2 |
| Pterygia fusion syndrome 1A | MYH3 |
| Pulmonary surfactant metabolism dysfunction 2 | SFTPC |
| Pulmonary surfactant Metabolism Dysfunction 3 | ABCA3 |
| Pulmonary Vasculopathy | VEGFD |
| Pyridoxamine 5'-phosphate oxidase deficiency | PNPO |
| Pyridoxine-dependent epilepsy | ALDH7A1 |
| Pyruvate dehydrogenase E1-alpha deficiency | PDHA1 |
| Pyruvate kinase deficiency | PKLR |
| Recurrent fetal hydrops | WDR44 |
| Recurrent metabolic encephalomyopathic crises | TANGO2 |
| Recurrent neuropathy | PMP22 |
| Renal cell carcinoma 1 | MET |
| Renal cysts and diabetes syndrome | HNF1B |
| Renal Tubular Acidosis 2 | ATP6V1B1 |
| Renal tubular dysgenesis | ACE |
|  | AGT |
| Rennpenning syndrome | PQBP1 |
| Resistance to Insulin-like growth factor 1 | IGF1R |
| Restrictive dermopathy 1 | ZMPSTE24 |
| Retinitis pigmentosa 1 | RP1 |
| Retinitis pigmentosa 11 | PRPF31 |
| Retinitis pigmentosa 12 | CRB1 |
| Retinitis pigmentosa 2 | RP2 |
| Retinitis pigmentosa 20 | RPE65 |
| Retinitis pigmentosa 25 | EYS |
| Retinitis pigmentosa 3 | RPGR |
| Retinitis pigmentosa 37 | NR2E3 |
| Retinitis pigmentosa 4 | RHO |
| Retinoblastoma | RB1 |
| Retinoschisis | RS1 |
| Rett syndrome | MECP2 |
| Rh system | RHD |
| Rhabdoid tumor predisposition syndrome 1 | SMARCB1 |
| Rhizomelic chondrodysplasia punctata-1 | PEX7 |
| Rhizomelic chondrodysplasia punctata-2 | GNPAT |
| Right atrial isomerism | GDF1 |
| Rigidity and multifocal seizure syndrome | BRAT1 |
| Ritscher-Schinzel Syndrome 2 | CCDC22 |
| Roberts-SC phocomelia syndrome | ESCO2 |
| ROSAH syndrome | ALPK1 |
| Rothmund-Thomson syndrome-2 | RECQL4 |
| Saethre-Chotzen syndrome | TWIST1 |
| Salla disease | SLC17A5 |
| Sandhoff disease | HEXB |
| Schaaf-Yang syndrome | MAGEL2 |
| Schwannomatosis-2 | LZTR1 |
| Seizures, cortical blindness, microcephaly syndrome | DIAPH1 |
| Sengers syndrome | AGK |
| Senior-Loken syndrome 6 | CEP290 |
| Senior-Loken syndrome 8 | WDR19 |
| Sensory neuropathy-1A | SPTLC1 |
| Severe combined immunodeficiency | RAG2 |
|  | RAG1 |
|  | IL2RG |
| Severe combined immunodeficiency T/B | JAK3 |
| Severe Combined Immunodeficiency, Athabascan | DCLRE1C |
| Severe congenital neutropenia 4 | G6PC3 |
| Severe junctional Epidermolysis bullosa 1B | LAMB3 |
| Severe junctional Epidermolysis bullosa 2B | LAMA3 |
| Short chain acyl-CoA dehydrogenase deficiency | ACADS |
| Short-rib thoracic dysplasia 11 with or without polydactyly | DYNC2I2 |
| Short-rib thoracic dysplasia 3 | DYNC2H1 |
| Short-rib thoracic dysplasia 5 with/without polydactyly | WDR19 |
| Short-rib thoracic dysplasia 8 with/without polydactyly | dync2i1 |
| Short-rib thoracic dysplasia 9 | IFT140 |
| Shwachman-Diamond syndrome 1 | SBDS |
| Sickle cell anemia | HBB |
| Sideroblastic anemia 1 | ALAS2 |
| Simpson-Golabi-Behmel syndrome-1 | GPC3 |
| Sjogren-Larsson Syndrome | ALDH3A2 |
| Smith-Lemli-Opitz syndrome | DHCR7 |
| Somatic Hepatoblastoma | APC |
| Sotos syndrome | NSD1 |
| Spastic ataxia, Charlevoix-Saguenay type | SACS |
| Spastic paraplegia 2 | PLP1 |
| Spastic Paraplegia 31 | REEP1 |
| Spastic paraplegia 3A | ATL1 |
| Spastic paraplegia 4 | SPAST |
| Spastic paraplegia 11 | SPG11 |
| Spastic paraplegia 47 | AP4B1 |
| Spastic paraplegia 52 | AP4S1 |
| Spastic paraplegia 54 | DDHD2 |
| Spastic paraplegia 57 | TFG |
| Spastic paraplegia 6 | NIPA1 |
| Speech-language disorder-1 | FOXP2 |
| Spherocytosis-1 | ANK1 |
| Spherocytosis-2 | SPTB |
| Spinal bulbar muscular atrophy | AR |
| Spinal muscular atrophy 1 | SMN1 |
| Spinocerebellar ataxia 1 | ATXN1 |
| Spinocerebellar ataxia 15 | ITPR1 |
| Spinocerebellar Ataxia 17 | TBP |
| Spinocerebellar ataxia 2 | ATXN2 |
| Spinocerebellar ataxia 29 | ITPR1 |
| Spinocerebellar ataxia 6 | CACNA1A |
| Spinocerebellar ataxia 7 | ATXN7 |
| Split-hand/foot malformation 3 | FBXW4 |
| Split-hand/foot malformation 6 | WNT10B |
| Spondyloarthropathy | HLA-B27 |
| Spondylocostal dysostosis 1 | DLL3 |
| Spondyloepimetaphyseal dysplasia | ACAN |
| Spondyloepimetaphyseal dysplasia-2 | KIF22 |
| Spondyloepimetaphyseal dysplasia-Krakow | SIK3 |
| Spondyloepiphiseal dysplasia | COL2A1 |
| Spondylometaphyseal dysplasia, Kozlowski type | TRPV4 |
| Stargardt Disease 1 | ABCA4 |
| Stickler syndrome-1 | COL2A1 |
| Stickler syndrome-2 | COL11A1 |
| Striatonigral degeneration | VAC14 |
| Stuve-Wiedemann Syndrome 1 | LIFR |
| Sudden cardiac failure | PPA2 |
| Sulfite oxidase deficiency | SUOX |
| Supravalvar aortic stenosis | ELN |
| Susceptibility to atypical hemolytic uremic syndrome 1 | CFH |
| Susceptibility to atypical hemolytic uremic syndrome 3 | CFI |
| Susceptibility to atypical hemolytic uremic syndrome 5 | C3 |
| Susceptibility to breast cancer | ATM |
|  | BRIP1 |
|  | BARD1 |
|  | CHEK2 |
|  | NBN |
| Susceptibility to breast cancer/pancreatic cancer 3 | PALB2 |
| Susceptibility to breast-ovarian cancer 3 | RAD51C |
| Hereditary breast-ovarian cancer 4 | RAD51D |
| Susceptibility to celiac disease 1 | HLA-DQA1 |
| Susceptibility to cutaneous malignant melanoma 2 | CDKN2A |
| Susceptibility to cutaneous malignant melanoma 8 | MITF |
| Susceptibility to intracerebral hemorrhage | COL4A1 |
| Susceptibility to Melanoma 10 | POT1 |
| Susceptibility to Myelodysplastic syndrome | GATA2 |
| Susceptibility to neural tube defects | TBXT |
| Susceptibility to Schwannomatosis 1 | SMARCB1 |
| Syndromic Diarrhea 3 | SPINT2 |
| TARP syndrome | RBM10 |
| Tay-Sachs disease | HEXA |
| T-cell lymphopenia, infantile, with or without nail dystrophy | FOXN1 |
| Thiamine metabolism dysfunction syndrome 2 | SLC19A3 |
| Thiamine metabolism dysfunction syndrome 5 | TPK1 |
| Thoracic aortic aneurysm 4 | MYH11 |
| Thrombocytopenia-absent radius syndrome | RBM8A |
| Thrombophilia due to protein C deficiency | PROC |
| Thrombophilia due to thrombin defect | F2 |
| Tooth agenesis 3 | PAX9 |
| Torsion dystonia-1 | TOR1A |
| Transient infantile liver failure | TRMU |
| Transthyretin-related amyloidosis | TTR |
| Treacher Collins syndrome 1 | TCOF1 |
| Treacher Collins syndrome 2 | POLR1D |
| Trichorhinophalangeal syndrome-1 | TRPS1 |
| Trifunctional protein deficiency | HADHB |
| Tuberous sclerosis 1 | TSC1 |
| Tuberous sclerosis 2 | TSC2 |
| Tubulointerstitial kidney disease 1 | UMOD |
| Tubulointerstitial kidney disease 2 | MUC1 |
| Tumor predisposition syndrome | BAP1 |
| Tyrosinemia-1 | FAH |
| Usher syndrome-1 | MYO7A |
| Usher syndrome-1D | CDH23 |
| Usher syndrome-1F | PCDH15 |
| Usher syndrome-2A | USH2A |
| Usher Syndrome-3A | CLRN1 |
| Van der Woude syndrome 1 | IRF6 |
| Van Maldergem syndrome 1 | DCHS1 |
| Ventriculomegaly with cystic kidney disease | CRB2 |
| Very long chain acyl-CoA dehydrogenase deficiency | ACADVL |
| Vici syndrome | EPG5 |
| von Hippel-Lindau syndrome | VHL |
| von Willebrand disease 2A | VWF |
| von Willebrand disease 3 | VWF |
| Waardenburg syndrome-1 | PAX3 |
| Waardenburg syndrome-2A | MITF |
| Waardenburg syndrome-2E | SOX10 |
| Waardenburg Syndrome-4 | EDNRB |
| Wagner syndrome 1 | VCAN |
| Warburg micro syndrome 1 | RAB3GAP1 |
| Warsaw breakage syndrome | DDX11 |
| Whim Syndrome 1 | CXCR4 |
| Wieacker-Wolff syndrome | ZC4H2 |
| Wilms tumor-1 | WT1 |
| Wilson disease | ATP7B |
| Wiskott-Aldrich syndrome | WAS |
| Xeroderma pigmentosum B | ERCC3 |
| Xeroderma pigmentosum D | ERCC2 |
| Xeroderma pigmentosum G | ERCC5 |
| Xp microdeletion syndrome | Xp |
